# Metabolic Impact of Flavonoids Consumption in Obesity: From Central to Peripheral

**DOI:** 10.3390/nu12082393

**Published:** 2020-08-10

**Authors:** Viviana Sandoval, Hèctor Sanz-Lamora, Giselle Arias, Pedro F. Marrero, Diego Haro, Joana Relat

**Affiliations:** 1 Department of Nutrition, Food Sciences and Gastronomy, School of Pharmacy and Food Sciences, Food Torribera Campus, University of Barcelona, E-08921 Santa Coloma de Gramenet, Spain; vivianapazsandovals@gmail.com (V.S.); h.sanz.lamora@gmail.com (H.S.-L.); giselle.arias@upr.edu (G.A.); pedromarrero@ub.edu (P.F.M.); 2 Institute of Nutrition and Food Safety of the University of Barcelona (INSA-UB), E-08921 Santa Coloma de Gramenet, Spain; 3 Institute of Biomedicine of the University of Barcelona (IBUB), E-08028 Barcelona, Spain; 4 CIBER Physiopathology of Obesity and Nutrition (CIBER-OBN), Instituto de Salud Carlos III, E-28029 Madrid, Spain

**Keywords:** non-alcoholic fatty liver disease, obesity, flavonoids, lipid metabolism, metabolic regulation, adipose tissue, brain

## Abstract

The prevention and treatment of obesity is primary based on the follow-up of a healthy lifestyle, which includes a healthy diet with an important presence of bioactive compounds such as polyphenols. For many years, the health benefits of polyphenols have been attributed to their anti-oxidant capacity as free radical scavengers. More recently it has been described that polyphenols activate other cell-signaling pathways that are not related to ROS production but rather involved in metabolic regulation. In this review, we have summarized the current knowledge in this field by focusing on the metabolic effects of flavonoids. Flavonoids are widely distributed in the plant kingdom where they are used for growing and defensing. They are structurally characterized by two benzene rings and a heterocyclic pyrone ring and based on the oxidation and saturation status of the heterocyclic ring flavonoids are grouped in seven different subclasses. The present work is focused on describing the molecular mechanisms underlying the metabolic impact of flavonoids in obesity and obesity-related diseases. We described the effects of each group of flavonoids in liver, white and brown adipose tissue and central nervous system and the metabolic and signaling pathways involved on them.

## 1. Introduction

Overnutrition and unhealthy diets together with physical inactivity cause an impairment in the metabolic homeostasis that lead to the development of pathologies such as obesity, type 2 diabetes, cardiovascular diseases (CVD) and more recently this kind of lifestyle has also been linked to neuroinflammation and neurodegenerative diseases [1,2,3,4,5].

The metabolic syndrome (MetS) is the medical term used to define the concomitance in an individual of some of the following alterations: hyperglycemia and/or insulin resistance, arterial hypertension, dyslipidemia and central or abdominal obesity [6]. It is currently one of the main public health problems worldwide and its incidence increases significantly each year, affecting almost 25% of the adult population today and has been directly associated to a greater risk of suffering from CVD or type 2 diabetes among others [3].

Obesity is one of the most important trigger for many of the other alterations include in the MetS. Obesity is essentially caused by an imbalance between energy intake and energy expenditure that initially causes an expansion of the white adipose tissue (WAT) to store the overfeed as triglycerides (TG). Some evidences indicate that at some point, WAT fails to adequately keep the surplus of nutrients and together with an insufficient differentiation of new adipocytes lead to an off-WAT accumulation of lipids in peripheral relevant organs. This ectopic accumulation of lipids causes lipotoxicity that may be, at least in part, responsible of the metabolic obesity-related metabolic dysfunctions [7]. It seems obvious that defects in WAT functionality together with peripheral lipotoxicity are the key points in the onset of metabolic syndrome (MetS) [8]. Looking for a way to restore lipid homeostasis and reduce lipotoxicity but also to diminish adipose tissue inflammation and macrophage infiltration many research groups are focused on identifying specific dietary patterns or foods capable to counteract these effects to finally revert obesity and its comorbidities.

Furthermore, it has been described that long-term hyperglycemia and diabetes complications induce impairments in the hippocampal synaptic plasticity as well as cognitive deficits [9] and increase the risk for Alzheimer disease [10,11] and depressive illness [12]. On the other side, diet-induced hypothalamic inflammation and mitochondrial dysfunction result in the onset and development of obesity and related metabolic diseases. It has been shown that, in rats, high fat diet (HFD) induces metabolic inflammation in the central nervous system (CNS), particularity in the hypothalamus [13].

The prevention of MetS and obesity is primary based on the follow-up of a healthy lifestyle, which includes, among other recommendations, a healthy diet. In this context, the Mediterranean Diet (DietMEd) has shown beneficial effects on the prevention and treatment of MetS and obesity by reducing chronic low-grade inflammation, improving endothelial function and reducing cardiovascular risk [14,15,16]. The study of Prevention with Mediterranean Diet (Predimed) has shown that high adherence to this nutritional profile is effective in the primary and secondary prevention of CVD, diabetes and obesity [17,18,19,20,21,22,23,24]. DietMed is characterized by a high consumption of foods rich in bioactive compounds such as polyphenols to whose have been attributed a large part of the health effects of this diet [18,23,25,26,27,28].

In this review, we have summarized the current knowledge on the metabolic effects of a specific group of polyphenols, the flavonoids, and the molecular mechanisms underlying these effects.

Concretely, the main goal of the present work is to describe the molecular mechanisms underlying the anti-obesity effects of flavonoids in three target organs/tissues: liver, adipose tissues (WAT and brown adipose tissue (BAT)) and central nervous system (CNS).

We choose a high variety of obesity models, sources and doses of flavonoids to identify the metabolic and signaling pathways involved in the effects of each subclass of flavonoids (anthocyanins, flavanols, flavanones, flavonols, isoflavones, flavones and chalcones) in these tissues/organs. Only studies in humans and experimental approaches whit animal models from the last years have been included, thus avoiding cell culture experimental approaches except when relevant.

## 2. Polyphenols and Metabolism

Polyphenols are the most abundant phytochemicals in nature. They are widely distributed in fruits, vegetables, and highly present in foods like legumes, cocoa, some cereals as well as in some beverages, such as tea, coffee and wine [29]. Polyphenols are not essential nutrients for humans but research in nutrition, including epidemiological studies, randomized controlled trials, in vivo and in vitro assays with animal models and cell lines, has shown that long-term and acute intakes can have beneficial effects on weight management and chronic diseases such as CVD, obesity, type 2 diabetes, the onset and development of some cancers and cognitive function [13,30,31,32,33,34,35,36,37].

The effects of polyphenols are directly related to their bioavailability. It is assumed that just the 5%-10% of the total dietary polyphenol intake is absorbed directly through the stomach and/or small intestine, the rest reaches the colon where they are transformed by the microbiota [38,39,40]. After being absorbed, polyphenols undergo phase I and II metabolism (sulfation, glucuronidation, methylation, and glycine conjugation) in the liver [29]. Polyphenol metabolites derived from liver metabolism may interact, among others, with adipose tissue, pancreas, muscle, and liver, where they exert their bioactivity.

Polyphenols have been divided in two main families: flavonoids and non-flavonoids, that are subdivided into several subclasses. For many years, the health benefits of polyphenols have been attributed to their anti-oxidant capacity as free radical scavengers. More recently it has been described that polyphenols activate other cell-signaling pathways that are not related to ROS production but rather involved in metabolic regulation [23,41].

### Flavonoids

Flavonoids are widely distributed in the plant kingdom when are used for vegetables for growing and defensing. They are structurally characterized by two benzene rings and a heterocyclic pyrone ring and based on the oxidation and saturation status of the heterocyclic ring flavonoids are grouped in seven different subfamilies (Table 1).

Flavonoids are abundant in food and beverages highly consumed by human population including fruits, vegetables, tea, cocoa or wine [42] and in global are the bioactive compounds more largely associated with a reduced risk of all-cause mortality, type 2 diabetes [43,44,45,46], CVD [36,47], obesity and its comorbidities such as non-alcoholic fatty liver disease (NAFLD) [48,49,50] and more recently they have been described as potential therapeutic agents against cognitive pathologies such as Alzheimer’s disease (AD) [42,51,52] or cerebrovascular alterations [47].

The molecular mechanisms underlying the beneficial effects of flavonoids have been widely studied and, in many cases, involved the activation of the AMP-activated protein kinase (AMPK). AMPK is a key enzyme for the control of lipid metabolism and adipogenesis. AMPK phosphorylation and activation promote catabolic processes such as FAO, glucose uptake, or glycolysis as well as inhibits anabolic pathways such as fatty acid synthesis or gluconeogenesis [53].

## 3. Anthocyanins

Anthocyanins are natural pigments and are responsible for the red-blue color of several flowers, fruits (mainly berries and grapes), roots, seeds (beans) but also of some leaves and cereal grains where they are found in low concentrations. Cyanidin, delphinidin, malvidin and their derivates are the most commonly studied anthocyanins [29,42,54,55,56].

Anthocyanins have shown antioxidant and anti-inflammatory properties but also positive effects in obesity and its comorbidities [57,58,59,60]. Several studies have demonstrated that the intake of anthocyanins by itself or of anthocyanins-rich foods such as berries is able to prevent CVD [61], to reduce body fat accumulation, to improve glucose tolerance/insulin sensitivity, to diminish the levels of fasting glucose, to control body weight in humans and rodents [57,59,62,63,64,65,66,67,68,69,70,71,72] and to increase energy expenditure and fatty acid oxidation (FAO) in mice and humans [59,73,74,75,76]. Globally, anthocyanins and anthocyanins-rich foods are able to improve metabolic homeostasis. More recently, anthocyanins have also revealed promising effects on cognitive function [51,77,78,79].

Part of the anthocyanins metabolic effects occur by regulating adipogenesis, increasing FAO, lipolysis, thermogenesis and mitochondrial biogenesis, regulating satiety and reducing lipogenesis in different tissues and organs and enhancing energy expenditure and body weight progression [74,75,76,80,81,82,83] Dietary supplementation with anthocyanins improves the lipid profile by favorably controlling the circulating levels of TG, total cholesterol, LDL-cholesterol and HDL-cholesterol [84].

### 3.1. Anthocyanins Improve the Metabolic Hemostasis in Obesity: The Liver Response

Non-alcoholic fatty liver disease (NAFLD) is characterized by an excessive accumulation of lipids in the livers. Its onset is closely related to obesity where an imbalance between fatty acids input and output causes initially a hepatic steatosis that can progress to NAFLD, non-alcoholic steatohepatitis (NASH), fibrosis, cirrhosis and in some cases hepatocarcinoma. Anthocyanins and anthocyanins-rich foods extracts or juices have demonstrated in several studies their ability to reduce the hepatic content of TG and lipids [85,86] and their capacity to modulate hepatic metabolism to protect against NAFLD [62,87,88,89]. Although in most of the published approaches performed with rodent models of obesity or NAFLD, anthocyanins or anthocyanin-rich fruits or extracts significatively reduced the hepatic lipid content and ameliorated the hepatic steatosis profile of these animals [88,90,91,92] some ineffective approaches have also been described [93,94,95].

The beneficial effects of anthocyanins in the liver have been linked to the activation of the AMPK, the upregulation of glycolytic and FAO genes and the downregulation of the gluconeogenic and lipogenic genes among others [70,71,72,96,97].

Mulberry anthocyanin extract administration to type 2 diabetic mice increased the activity of AMPK/peroxisome proliferator-activated receptor gamma coactivator 1 alfa (PGC1α)/p38 mitogen-activated protein kinase (MAPK) and reduced the activity of the acetyl-CoA carboxylase enzyme (ACC), a rate-limiting enzyme of fatty acid synthesis, and of the mammalian target of rapamycin (mTOR) that is involved in protein synthesis regulation and insulin signaling [96]. Similar effects were described in HFD-fed hamsters, where Mulberry water extracts exerted anti-obesity effects by inhibiting lipogenesis (downregulation of fatty acid synthase (FASN) and 3-hydroxy-3-methylglutaryl-coenzyme A (HMG-CoA) reductase) and upregulating PPARα and CPT1A [81]. On its side, honeyberry (*Lonicera caerulea*) extract (HBE) also decreased lipid accumulation in the liver of HFD-obese mice. HBE downregulated the hepatic expression of lipogenic genes such as *sterol regulatory element-binding protein-1 (Srebp-1c), CCAAT/enhancer-binding protein alpha (C/ebpα), Pparγ,* and *Fasn* as well as upregulated the mRNA and protein levels of CPT1a and PPARα, thus enhancing FAO. As mulberry anthocyanin extract, HBE treatment also increased the phosphorylation of AMPK and ACC thus activating and inhibiting these enzymes respectively [98]. On the other hand, in NAFLD-induced rats, blackberry extracts improved insulin sensitivity and dyslipidemia, ameliorated triglyceride and lipid peroxide accumulation and suppressed the mRNA expression of genes involved in fatty-acid synthesis (*Fasn* and *Srebp-1c*) [88]. Finally, purple sweet potato reduced the protein levels of FASN and of the cluster of differentiation 36 (CD36), inactivated the C/EBPβ, restored AMPK activity and increased the protein levels of CPT1a in livers of HFD-fed mice, thus indicating decreased lipogenesis and fatty acid uptake and enhanced FAO [62].

Regarding glucose metabolism, protein-bound anthocyanin compounds of purple sweet potato ameliorate hyperglycemia in obese and diabetic mice by regulating hepatic glucose metabolism. Anthocyanin compounds of purple sweet potato induced the hepatic protein levels of p-AMPK, glucose transporter type 2 (GLUT2), insulin receptor α (IRα), glucokinase (GK)*,* as well as the expression of *phosphofructokinase (Pfk)* and *pyruvate kinase (Pk),* while gluconeogenic genes, *glucose-6-phosphatase (G6Pase)* and *phosphoenolpyruvate carboxykinase (Pepck)* were downregulated [99]. Further, Saskatoon berry normalized liver expression of *Gk* and *glycogen phosphorylase* and increased *G6Ppase* in diet-induced MetS rats, thus suggesting that Saskatoon berry regulated glycolysis, gluconeogenesis and glycogenesis to improve MetS [100].

Although most of the experimental approaches have been done using anthocyanins-rich extracts, pure compounds have been also analyzed. Cyanidin-3-glucoside (C3G) administration to C57BL/6J obese mice fed a HFD and db/db mice diminished the triglyceride hepatic content and steatosis [73,101], through the blockade of the c-Jun N-terminal kinase activation (JNK) and the promotion of the phosphorylation and nuclear exclusion of the transcription factor Forkhead box protein O1 (FoxO1) [101].

All these data confirm the impact of anthocyanins and even in a more significative way of the anthocyanin-rich foods on metabolism. These effects can be added to their anti-inflammatory, antiapoptotic, pro-autophagic and antioxidant properties in steatotic livers [59,62,102,103,104].

### 3.2. Anthocyanins in Adipose Tissue: The Activation of BAT and the Browning of WAT

The impairment of adipose tissue function is strongly associated with the development of obesity and insulin resistance (IR). The activation of BAT and the browning in WAT are considered potential strategies to counteract the metabolic alterations linked to the obese phenotype. Both actions are mechanisms to increase the energy expenditure (EE) through the induction of lipolysis, FAO and thermogenesis and consequently efficient ways to reduce the ectopic lipid accumulation and the lipotoxicity [105,106,107,108].

Part of the beneficial effects of anthocyanins on diet-induced obesity are due to their impact on adipose depots. Anthocyanidins regulate lipolysis, FAO, lipogenesis and adipose tissue development [76,109,110,111]. They affected the adipokines secretion [112], modified the adipocytes-gene expression [33,113,114]. Moreover, anthocyanins are able to improve WAT functionality, to induce browning in WAT [33,57,82,115] or to increase the BAT mass or its activity [57,109,115], thus regulating energy expenditure [59,73]. Moreover, in WAT, anthocyanins ameliorate the obesity-associated inflammation [57,59,116].

In WAT, an anthocyanin-rich bilberry extract ameliorated hyperglycemia and insulin sensitivity through the activation of AMPK that resulted in an increase of the glucose transporter 4 (GLUT4) [72]. On its side, C3G-enriched *Aronia melanocarpa* extract reduced food intake and WAT weight in HFD-fed mice but also suppressed adipogenesis. These animals showed a downregulating in the expression levels of *C/ebp*α, *Srebp1c, Acc, ATP-citrate lyase, Pgc1α, Fasn,* and *adipocyte protein 2 (Ap2)* as well as in the circulating levels of leptin [111]. In the same way, in HFD-induced obese mice model, the dietary supplementation with maqui (*Aristotelia chilensis*) improved the body weight gain and glucose metabolism at least in part by modifying the expression of the *carbohydrate responsive element binding protein β* (*Chrebpβ*), *the fibroblast growth factor 21* (*Fgf21*) and *adiponectin* as well as of the lipogenic and FAO genes [82]. Globally, the maqui supplementation induced the browning of the subcutaneous WAT (scWAT) [82].

The induction of browning is a common phenotype in obese rodent models treated with anthocyanins or anthocyanins-rich foods. The thermogenic and mitochondrial markers were also increased in the inguinal WAT (iWAT) of high fat-high fructose (HF/HFD)-fed mice treated with C3G, thus indicating the browning of this adipose tissue depot and suggesting an increased heat production and energy expenditure (EE) [117]. In db/db mice, C3G and vanillic acid exerted similar effects: increased EE, limited weight gain and upregulated expression of *Ucp1* and other thermogenic and mitochondrial markers, thus indicating the induction of brown-like adipocytes development in the scWAT [73] or iWAT [115]. Freeze dried raspberry decreased WAT hypertrophy induced by HFD and promoted the browning of WAT as it is showed by a higher expression of beige markers such as *Ucp1, PR-Domain zinc finger protein 16 (Prdm16), Cytochrome C, Cell death inducing DFFA like effector A (Cidea), and Fatty acid elongase 3(Elovl3),* elevated levels of PGC-1α and Fibronectin type III domain-containing protein 5 (FNDC5)/irisin, and an activation of the AMPK/Sirtuin 1 (SIRT1) pathway [33]. AMPK and Sirt1 are important sensors of the energy status that together with PGC-1α regulate energy homeostasis and stimulate FNDC5/irisin expression, thus inducing beige adipogenesis [118]. The regulation of adipogenesis through the AMPK/SIRT1 pathway has also been described in HFD fed mice treated with maize extract rich in ferulic acid and anthocyanins [119].

In WAT, anthocyanins and anthocyanin-rich foods also improve the inflammatory profile. The administration of a black soybean testa extracts (BBT) to diet-induced obese mice decreased fat accumulation, and the expression of *Acc* and *C/ebpα* and increased the levels of lipolysis proteins such as lipoprotein lipase (LPL), hormone-sensitive lipase (HSL) in mesenteric fat but also showed anti-inflammatory effects [109]. Similar effects were observed in humans where the administration of BBT to overweight or obese individuals decreased the abdominal fat measured as waist and hip circumference and improved the lipid profile [110]. The anti-inflammatory effects have been also achieved with sweet cherry anthocyanins and blueberry (*Vaccinium ashei)* anthocyanins. These anthocyanins reduced the body weight gain, the size of adipocytes and the leptin secretion in HFD-fed mice but also expression of *Il-6* and *Tnfa* genes, thus indicating an amelioration of the deleterious effects of a HFD [114,120].

Besides their effects on WAT, anthocyanins and anthocyanins-rich food also impact on BAT where they promote its activity. In high fructose/HFD-fed animals, besides inducing the browning of WAT, C3G attenuated the development of obesity by promoting the tremorgenic capacity of BAT. C3G upregulated the expression of thermogenic markers such as *Ucp1,* induced the mitochondrial biogenesis and function and finally increased the EE [117]. In db/db mice, C3G and vanillic improved cold tolerance and enhanced BAT activity and induced mitochondrial biogenesis. In BAT, anthocyanin and anthocyanin-rich foods upregulated the expression of thermogenic markers (*Ucp1, Prdm16, Cidea…)*, lipid metabolism *(Cpt1a, Hsl, adipose triglyceride lipase (Atgl)),* mitochondrial markers *(mitochondrial transcription factor A (Tfam), Nuclear Respiratory Factor 1* and *2 (Nrf1* and *Nrf2)…)* and transcriptional regulators or coactivators of these processes (*Pparα*, *Pgc1β*, *Pgc1α…)* [73,115].

### 3.3. In the Central Nervous System (CNS) Anthocyanins Have Been Related to Neuroprotective Effects as Well as in Feeding Behavior

The neuroprotective activity of anthocyanins has been widely evidenced in several epidemiological studies and their potential for the prevention of many neurodegenerative diseases such as Parkinson’s disease (PD) and Alzheimer’s disease (AD) has been suggested [77,78]. The neuroprotective effects of anthocyanins and C3G correlate with the regulation of molecules upstream of nitric oxide (NO) production, neuroinflammatory response and oxidative stress [79,121,122,123].

It has been demonstrated that C3G and malvidin 3-O-glucoside (M3G) inhibited the hyperphosphorylation of Tau protein in Alzheimer’s disease [124] and berries supplementation have shown neurocognitive benefits in older adults at risk for dementia with mild cognitive impairment [125]. Recent studies highlighted an anti-depressive effect of a maqui-berry extract in a mouse model of a post-stroke depression. In this case the maqui effects were associated to its antioxidant capacity [126]. Otherwise, anthocyanins extracted from dried fruits of *Lycium ruthenicum Murr* have demonstrated a protective role in cerebral ischemia/reperfusion injury in rats [127] by inhibiting cell apoptosis and reducing edema and inflammation.

Besides their role in neuroprotection, anthocyanins modulate the feeding behavior. In rats, anthocyanins from black soybean increase the expression of the gamma-aminobutyric acid B1 receptor (GABAB1R) and decrease the expression of neuropeptide Y (NPY) in the hypothalamus, thus modulating the food intake behavior/body weight control. The upregulation of GABABR1 is followed by a decrease of the activated protein kinase A (PKA) and the phosphorylated cAMP-response element binding protein (CREB), both located downstream of GABAR1 [83]. In a similar way, the administration of an anthocyanin-rich black soybean testa (Glycine max (L.) Merr.) to diet-induced obese mice decreased food intake [109].

## 4. Flavanols

Flavanols are present in cocoa, tea, red wine, beer and several fruits such as grapes, apricots, apples where they are responsible for their astringency [128]. Flavanols exist as monomers named catechins or as polymers named proanthocyanins. The monomeric forms include: catechin (−)-epicatechin (EC), (−)-epigallocatechin gallate (EGCG), (−)-epigallocatechin (EGC), and (−)-epicatechin gallate (ECG). The proanthocyanins, also known as tannins, are more complex structures (dimers, oligomers, and polymers of catechins) and can be transformed to anthocyanins [29]. Like other flavonoids, flavanols are absorbed between the small intestine and the colon depending on their physicochemical properties and structure [129].

Flavanols possess a health claim related to their role in maintaining the elasticity of blood vessels that was approved in 2014 by the European Food Safety Authority (EFSA) [130].

In humans and animal models, flavanols or flavanols-rich foods (mainly, cocoa or tea derivates) have demonstrated the ability to reduce body weight, decrease waist circumference and fat percentages, improve glucose metabolism in individuals with type 2 diabetes, obesity or MetS and increase energy expenditure [75,131,132,133,134,135,136,137,138,139]. One of the most described molecular mechanism underlying theses effects are the activation of the AMPK enzyme [140].

Due to the high amount of publications including flavanols and metabolism we just included a representative group of the most recently published and the ones that deepen more on the molecular mechanisms underlying the beneficial effects of flavanols.

### 4.1. Flavanols Improve Hepatic Steatosis and Glucose/Lipid Metabolism in Obesity Models

In humans and several rodent models of obesity, flavanols have been able to improve blood lipid profile and protect liver from excessive fat deposition and hepatic steatosis [136,141,142,143,144,145,146]. These effects have been related mostly with an activation of the AMPK and the protein kinase B (PKB/Akt) pathways that finally lead to the suppression of lipogenesis by modulating the expression of *Srebp1c*, *cAMP-response element-binding protein regulated transcription coactivator 2 (Crtc2),* and *stearyl coenzyme A dehydrogenase-1 (Scdh1)* or the activity of ACC, the inhibition of gluconeogenesis by affecting the levels of *PepcK* and *G6pase* and the increment of FAO by increasing the *Cpt1a* levels. Moreover, flavanols are able to improve cholesterol homeostasis through the regulation of several enzymes from the cholesterol synthesis and bile acids metabolism apart from the modulation of the mRNA expression of apolipoprotein B100 and ATP-binding cassette transporter A1. Most of the approaches included have been done using tea extracts or cocoa flavanols but other extracts with a more diverse composition of flavonoids have been also described in this section [137,143,147,148,149,150,151].

Theabrownin from Pu-erh tea in combination with swinging improved serum lipid profile and prevent development of obesity and insulin resistance in rats fed a high-fat-sugar-salt diet and subjected to a 30-min daily swinging. A transcriptomic analysis in the liver indicated that theabrownin together with exercise activated circadian rhythm, PKA, AMPK, and insulin signaling pathway, increased the levels of cAMP and accelerated the consumption of sugar and fat [142]. Similar results were obtained with HFD-fed mice supplemented with Yunkang green tea and subjected to treadmill exercise. These animals showed a reduction in the body weight gain and liver weight, a lower level of blood glucose, serum total cholesterol (TC), TG, insulin and ALT and an improvement in the fatty liver and hepatic pro-inflammatory profile compared to HFD group. Supplemented and exercised-animals showed a downregulation of the lipid synthesis genes (*Srebp1c, Fasn, Acc),* and an improvement of the hepatic insulin signaling [143].

Furthermore, in obese Zucker rats fed with a HFD and treated with green tea polyphenols a significant reduction on fasting insulin, glucose and lipids and an improvement of the NAFLD were observed. Livers of treated rats had lower levels of alanine aminotransferase (ALT) and aspartate aminotransferase (AST), of inflammatory markers and of TG content and exhibited less lipid droplets. These improvements have been related to an activation of the AMPK pathway and the inhibition of the hepatic lipogenesis (higher levels of the inactive p-ACC and lower levels of SREBP1c) [152]. These effects on lipid metabolism were also observed after the administration of Benifuuki (a tea that contains methylated catechins such as epigallocatechin-3-O-(3-O-methyl) gallate (EGCG3′’Me) to high fat/high sucrose diet-fed mice. Benifuuki treatment lowered the levels of TG and NEFA in serum and liver and reduced the expression of hepatic lipogenic genes (*Srebp-1c, Acc1, Fasn* and *Stearoyl-CoA desaturase 1(Scd1*)) [153]. In parallel the use of *Euterpe oleracea* Mart.-derived polyphenols, known by the popular name of açai and rich in catechin and polymeric proanthocyanins, when administered to HFD-fed mice [154] or a pistachio-diet supplementation to diet-induce obese mice exhibited similar impact on lipid metabolism and gene expression modulation [150].

Finally, Oliogonol, a flavanol-rich lychee fruit extract, significantly reduced hepatic lipid content (less lipid droplets and ballooning by downregulating the *Pparγ* and, *Srebp1c* mRNA levels [155] probably via the inhibition of the mTOR activity promoted by the activation of the AMPK enzyme [156]. Moreover, oligonol improved hepatic insulin sensitivity by reducing the phosphorylation of glycogen synthase kinase 3a (GSK3a) and the phosphatase and tension homologue (PTEN) in HFD-induced obese mice [155] as well as inhibiting the mTOR/S6K cascade. The activation of the mTOR/S6K phosphorylates and desensitizes the insulin receptor substrate 1 (IRS1) [157]. In a similar way, GC-(4→8)-GCG, a proanthocyanidin dimer from *Camellia ptilophylla* improved hepatic steatosis and hyperlipidemia in HFD-induced obese mice [158].

Besides on hepatic lipogenesis, tea extracts also impact in FAO. The administration of tea water extracts from green tea, yellow tea, white tea, black tea, raw pu-erh tea and oolong tea decreased TG and total cholesterol levels in serum and liver as well as the hepatic lipid content. Supplemented animals displayed less lipid droplets, the activation of the AMPK and the upregulation of the *Cpt1a* together with the inhibition of the FASN enzyme. These treatments also reduced the inflammation profile linked to HFD [149]. Similar results were obtained with grape seed procyanidin B2 (GSPB2) and a polyphenol extract from *Solanum nigrum* that contains among other different catechins. In db/db mice, GSPB2 decreased body weight and improved the lipid profile in serum (TG, total cholesterol and free fatty acids (FFA)) but also reduced hepatic lipid droplets and TG accumulation. The proposed mechanism implied the AMPK activation, the ACC phosphorylation and *Cpt1a* overexpression, thus inhibiting FA synthesis and increasing FAO [159]. In a similar way, the *Solanum nigrum* polyphenol extract inhibited lipogenesis and enhanced FAO (upregulation of *Cpt1a* and P*parα*) through the AMPK cascade [151].

In different animal models of obesity and insulin resistance, EGCG has shown the capacity to improve glucose homeostasis, to inhibit gluconeogenesis, FA and cholesterol synthesis and to increase FAO [147,148]. In HFD and STZ-induced type 2 diabetes, EGCG downregulated *Pepck* and *G6Pase* and inhibited SREBP1c, FASN and ACC1. The mechanism underlying these effects is not yet well understood but it has been suggested that EGCG would activate the PXR/CAR-mediated phase II metabolism that through a direct or indirect mechanism would suppress gluconeogenesis and lipogenesis [147]. Moreover, in HFD Wistar rats, EGCG diminished the liver weight, the hepatic hyperlipidemia, animals showed less lipid droplets, reduced serum levels of ALT and AST, TG, total cholesterol and better profile of LDL/HDL but also an ameliorated oxidative stress. In this case, EGCG activated SIRT1, FoXO1 and regulate SREBP2 activity to suppress hepatic cholesterol synthesis. These data point out the downregulation of SREBP2 expression under the SIRT1/FOXO1 signaling pathway as a mechanism to reduce the cholesterol content [148]. Furthermore, EGCG also decreased bile acid reabsorption, which decreased the intestinal absorption of lipids [160]. In the same way, EC administered to a high-fat high cholesterol diet rats reduced serum levels of total cholesterol, LDL and TG while increased HDL [161]. Moreover, EC intake also reduced serum levels of ALT and AST enzymes, the lipid peroxidation and the pro-inflammatory cytokines levels, thus indicating an improvement in the liver functionality. The proposed mechanism of EC included the downregulation of the nuclear receptor liver-X-receptor (LXR), the FASN enzyme and the SIRT1 protein but also the blockade of the Insig-1-SREBP-SCAP pathway that drives the SREBP2 maturation [161].

### 4.2. Flavanols in Adipose Tissue: Less Adiposity and More Energy Expenditure: The Browning Effect

In humans, some studies described the capacity of green tea to reduce body weight and abdominal fat accumulation [162,163], influence on the body fat mass index, waist circumference, total fat mass and energy expenditure through the induction of browning or BAT activity [164,165,166] but also to regulate ghrelin secretion and adiponectin levels, to control appetite and decrease nutrient absorption [135,167].

In rodents, the administration of grape seed-derived proanthocyanins to Wistar rats reduced the body weight by limiting food intake and activating EE in scWAT [168] and it has been widely described that in rodent models of obesity, flavanols are able to affect the lipid metabolism of WAT and BAT. Global effects of flavanols in adipose tissues lead to a decrease in adiposity, specially of the WAT depots and in adipocyte size by reducing adipogenesis, the release of adipokines such as leptin and resistin, the modulation of lipid metabolism and the induction of browning [153,155,158,169,170,171,172,173,174]. In BAT, flavanols caused the activation of thermogenesis and FAO [172,173,174,175,176].

As has been mentioned before, in WAT, flavanols modified lipid metabolism. EGCG reduced the expression of genes related with *de novo* lipogenesis (*Acc1, Fasn, Scd1, C/ebpβ, Pparγ* and *Srebp1c*), increased the expression of genes involved in lipolysis (*Hsl*) and lipid oxidization (*Pparα*, *Acetyl-CoA oxidase (Acox)2,* and medium-chain acyl-CoA dehydrogenase (*Mcad)*) in epididymal (eWAT) and scWAT and highly upregulated the expression of delta-9 desaturase, the enzyme responsible to convert saturated fatty acids to monounsaturated [177]. The activation of the AMPK in HFD-EGGC-treated mice indicated that at least in part the changes in lipid metabolism observed were due to the AMPK phosphorylation [177]. In scWAT, although EGCG increased lipolysis (*Hsl)* and FAO (*Cpt1a)* [168,178], some lipogenic genes (*Acc1, Fasn, Scd1, Pparγ,* and *Srebp1*) has been detected upregulated at the mRNA level but no at protein level [178]. These data suggested that EGCG might have different effects in scWAT and eWAT. Finally, pistachio-diet supplementation to diet-induce obese mice also ameliorated the HFD-induced expression of *Srebp1c*, *Pparγ*, and *Fatp* [150].

Besides its effects in the liver, the GC-(4→8)-GCG inhibited the expansion of all WAT depots in HFD fed mice. Adipocytes from eWAT were smaller and some of the main adipocyte-associated transcription markers were downregulated (*Srebp1c, C/ebpα and Pparγ*), thus indicating a better WAT functionality [158]. The GC-(4→8)-GCG-supplemented mice showed an upregulation of the adiponectin and a downregulation of the leptin mRNA levels as well as an improved inflammatory profile with less macrophage infiltration [158].

Regarding the browning effect of flavanols it has been published that EC increased mitochondrial biogenesis, fatty acid metabolism and upregulated the expression of BAT-specific markers (*Prdm16*, *Dio2*, *Ucp1* and U*cp*2) in WAT in a way that depends on phosphorylation and deacetylation cascades [170]. The authors demonstrated that EC supplementation upregulated the mitochondrial related proteins p-SIRT1, SIRT1, SIRT3, PGC1α, PPARγ, TFAM, NRF1, NRF2, complex II, IV and V and mitofilin [170]. In a similar way, a polyphenolic extract from green tea leaves (GTE) ameliorated the body weight gain caused by a HFD with no changes in calorie intake but reducing the adiposity and the adipocyte size in WAT and BAT. GTE supplementation induced BAT markers in scWAT (higher mRNA levels of *Pgc1α, Cbp/p300-interacting transactivator 1 (Cited1)* and *Prdm16* and of UCP1 protein) and reduced HFD-induced whitening in BAT (lower expression of adipogenic markers *C/ebpα* and *Ap2* and upregulation of *Pgc1α* and *vascular endothelial growth factor-A(165)* (*Vegfa165*)) [171]. These animals also showed an improvement in the inflammatory profile in scWAT and BAT. Finally, a Grape pomace extract (GPE) showed the capacity to induce browning (upregulation of *Pgc1α, Pparγ, Prdm16* and *Ucp1*) in the eWAT of HFD-fed rats [179,180].

Besides tea extracts also cacao components are able to induce browning and BAT activation. Concretely, theobromine alleviated diet-induced obesity in mice by inducing a brown-like phenotype in the iWAT and activated lipolysis and thermogenesis in BAT. In HFD fed mice theobromine inhibited phosphodiesterase-4 (PDE4D) activity in adipose tissue, thus increasing β3-adrenergic receptor (AR) signaling pathway and EE [172]. The inhibition of PDE increases the cellular levels of cAMP levels thus activating the β-AR cascade and finally PKA and UCP1 activity [181].

The capacity of flavanols on activating BAT has been described even with a single dose of a flavanol mixture that included catechins and B type procyanidins or by administering individual components by itself [182]. In these animals, *Ucp1* mRNA expression in BAT and levels of catecholamines in plasma were significantly increased via SNS stimulation but with varying efficacy depending on the stereochemical structure of flavanols [182]. It should be noted that prolonged ingestion of a catechin-rich beverage increased the BAT density with a decrease in extramyocellular lipids in humans [183]. EGCG-supplemented diet-induced obese mice exhibited higher body temperature and more mitochondrial DNA (mtDNA) content in BAT together with an upregulation of the genes related to fatty acid metabolism, thermogenesis and mitochondrial biogenesis (*Ucp1, Ucp2, Prdm16, Cpt1β, Pgc-1α, Nrf1,* and *Tfam*) [184,185] and a downregulation of *Acc*. These effects have been related to an increased activity of the AMPK in BAT [184].

Thermogenesis can also be induced by a polyphenol-rich green tea extract (PGTE) through a mechanism that depends on adiponectin signaling. The treatment with this extract reversed part of the obesity phenotype in WT mice but no in adiponectin KO mice (AdipoKO). PGTE treatment increased EE, BAT thermogenesis, and promoted browning phenotype in the scWAT of WT mice but these effects were blunted in AdipoKO mice [176].

Some data regarding BAT activation by catechins in humans have also described. Different approaches have been done to demonstrate the effects of green tea extract and caffeine over thermogenesis and body weight [186,187]. Short- and long-term effects have been studied with different results and effectiveness but suggesting that catechins and caffeine may act synergistically to control body weight and induce thermogenesis [175,188]. It has been proposed that the thermogenic response to green tea extracts or its components would be mediated, in BAT, by the direct stimulation of the β-adrenergic receptor (β-AR) cascade through the inhibition of the enzyme catechol-O-methyl transferase (COMT), which degrades catecholamines. On its side, caffeine inhibited PDE, thus inducing a sustained activation of the PKA and its downstream cascade [175].

### 4.3. Flavanols Consumption Induces Energy Expenditure in Peripheral Organs through the Sympathetic Nervous System Activation

Part of the anti-obesity effects of flavanols have been also related to their influence on sympathetic nervous system (SNS) activity. The SNS activation by green tea catechins (GTC) has been associated to their capacity to inhibit COMT. The inhibition of COMT leads to a prolonged activation of the sympathetically-response and of the β-adrenergic cascade that produces cAMP and the activation of the PKA. Caffeine, in turn, is able to inhibit the PDE activity which drives to a sustained activation of the PKA and its downstream response [175]. Then, both effects act synergistically to increase EE, lipolysis and FAO as has been described in the above sections. Some other mechanisms to describe the anti-obesity effects of flavanols include the modulation of food intake. It has been demonstrated that grape-seed proanthocyanins extract (GSPE) reduced food intake in rats fed a cafeteria diet. These animals showed an activation of the STAT3 protein which upregulated the *pro-opiomelanocortin (Pomc)* expression, thus improving the leptin resistance [189].

Moreover, GSPE supplementation reduced the neuroinflammation and increased the expression of SIRT1 [189]. Flavanols has been described as active molecules against diet-induced neuroinflammation. The induction of neuroinflammation and cognitive impairment in rats by feeding them with a high salt and cholesterol diet (HSCD) could be in part reversed by the treatment with different doses of an enriched-tannins fraction of the Indian fruit *Emblica officinalis.* Treatment with this tannin-enriched gooseberry reversed the HSCD-induced behavioral and memory disturbances, neuronal cell death and reduced the levels of cognitive impairment markers. [190]. In the same way, it has been published that, in mice, EGCG attenuated the neuronal damage and insulin resistance caused by a high fat/high fructose diet (HF/HFD). In this case, EGCG upregulated the IRS-1/AKT and the extracellular-signal-regulated kinase (ERK)/CREB/Brain-derived neurotrophic factor (BDNF) signaling pathways. In longer nutritional interventions with the HF/HFD, EGCG was capable to inhibit the MAPK and NF-κB pathways, as well as the expression of inflammatory mediators, such as TNF-α to reverse the neuroinflammation [191]. Similar results were obtained with EGCG-HFD dietary supplementation. The authors demonstrated that EGCG ameliorated the HFD-induced obesity in part by attenuating hypothalamic inflammation through the inhibition of NF-kB and Signal transducer and activator of transcription 3 (STAT3) phosphorylation, as well as the expression and release of inflammatory cytokines, such as TNF-a, IL-6, and IL-1b [185].

Finally, EGCG alleviated part of the cognitive deficits in a mixed model of familial Alzheimer’s disease (AD) and type 2 diabetes mellitus (T2DM). The AD mice model APP/PS1 fed with a HFD showed an improvement in peripheral parameters such as insulin sensitivity but also in central memory deficits when treated with EGCG. Synaptic markers and CREB phosphorylation were increased because of an amelioration in the unfolded protein response (UPR) activity via a downregulation of the activation factor 4 (ATF4) levels. Moreover, EGCG decreased brain amyloid β (Aβ) production and plaque burden by increasing the levels of α-secretase (ADAM10) and reduced the neuroinflammation in these animals [192]. Finally, green tea extracts can modulate the redox status of the CNS in obese and lean rats [193].

## 5. Flavanones

Flavanones are a subfamily of flavonoids widely distributed in *citrus* fruits such as grape, tomatoes, and oranges and are the responsible of the bitter taste of their peel and of their juice. As other flavonoids, flavanones show strong health benefits due to its antioxidant activity but also exhibit antiviral, antimicrobial, antiatherogenic, anti-inflammatory antidiabetic and anti-obesity properties [45,48,75,194,195]. Flavanones are mainly found as aglycones or as glycosylated derivatives [196]. The most studied flavanones are hesperidin, naringenin but also eriodyctiol, isosakuranetin and taxifolin.

Hesperidin and its aglycone, hesperetin are found in citrus fruits, such as limes and lemons, tomatoes and cherries and have demonstrated antidiabetic, neuroprotective, antiallergic, anti-inflammatory anticarcinogenic besides their well-established antioxidant capacity [45,197] Naringenin and its aglycone naringin are found to be more abundant in citrus fruits such as grapefruit orange, lemon but also in tomatoes. Naringenin and derivates have been associated with beneficial effects in cardiovascular diseases, osteoporosis, cancer and have showed anti-inflammatory, antiatherogenic, lipid-lowering, neuroprotective, nephroprotective, hepatoprotective and antidiabetic properties [198,199].

### 5.1. Flavanones-Dietary Supplementation Ameliorates the NAFLD in Humans

Frequently, liver diseases are initiated by oxidative stress, inflammation and lipid accumulation that lead to an excessive production of extracellular matrix followed by a progression to fibrosis, cirrhosis and hepatocellular carcinoma [200]. In the last years, several studies have demonstrated the capacity of different flavanones to ameliorate liver diseases.

To analyze the positives effects of flavanones in liver different approaches have been used. Some authors worked with hepatic chemical-induced damage being the most used the streptozotocin injection to mice or rats [199,201]. Other authors induced liver damage with diet [199] or worked with genetically obese models. Although flavanones demonstrated positive effects in the different approaches, in this review we focused on the experimental approaches where the liver disease has been induced by diet or where genetically obese-models has been used. Experiments with naringenin, hesperidin and eriodyctiol has been done to evaluate the impact of this flavanones’ consumption in NAFLD or liver steatosis.

Naringenin has showed the capacity to restore the activities of liver hexokinase, PK, G6Pase and Fructose 1,6-bisphosphatase from rats fed a high fructose diet to levels similar to healthy non-diabetic animals [202]. In this animal model, naringenin also enhanced liver protein tyrosine kinase (PTK), while reduced protein tyrosine phosphatase (PTP) activity [202]. In addition, administration of naringenin to HF/HSD-fed rats increased the protein levels of PPARα, CPT1a and UCP2 [203]. In a similar way, naringenin increased FAO and the AMPK activity in HFD fed mice where ameliorated the metabolic alterations caused by diet [204]. Similar results were obtained in high-fat/high-cholesterol (HFHC) fed Ldlr -/- mice. In lean Ldlr -/- mice, naringenin induced weight loss and reduce calorie intake, enhanced EE and increased hepatic FAO by upregulating *Pgc1α, Cpt1a and Hsl*, thus indicating that naringenin is also effective in non-obese models [195]. In HFD fed Ldlr -/-, naringenin increased FAO and reduced lipogenesis. Hepatic *Srebp1c* and *Acox1* mRNA levels were downregulated, while *Fgf21, Pgc1α, and Cpt1a* were upregulated by naringenin [205]. Later on, it was published that naringenin prevented obesity, hepatic steatosis, and glucose intolerance in an FGF21-independent way [206]. More recently, it has been described that in obese-mice naringin decreased hepatic liver content (TG and total cholesterol) and activated the AMPK enzyme resulting in reduced expression and protein levels of liver SREBP1C, SREBP2, but increased LDLR. Moreover, these mice showed reduced plasma levels of proprotein convertase subtilisin/kexin type 9 (PCSK9), leptin, insulin, and LDL-C compared to obese non-treated mice [207].

Besides naringenin, naringin and hesperidin effects in liver have also been evaluated. Hesperidin and naringin supplementation in *db/db* and *ob/ob* mice regulated hepatic gluconeogenesis and glycolysis, as well as lipid metabolism [208]. Hesperidin stimulated PPARγ, increased the hepatic GK activity and glycogen concentration and reduced the hepatic levels of *Glut2* as well as increased the expression of *Glut4* in WAT [46,208,209]. Moreover, hesperidin prevented hepatic steatosis in western diet-fed rats by preventing the upregulation of lipogenesis-related genes *Srebf1,* and *Scd1* caused by Western diet and the downregulation of *Ppar*α and *Cpt1a* expression and CPT1a protein levels [210]. Most of these effects were blunted when hesperidin is combined with capsaicin [210].

In diet-induced obese mice treated with neohesperidin the expression and secretion of FGF21 and the activity of the AMPK/SIRT1/PGC-1α axis were improved [211]. Treatment with neohesperidin improved the steatotic state (less and smaller lipid droplets), reversed the downregulation of hepatic *Pparα* levels while increased the levels of the hepatic *Fgf21* expression and its plasma levels. Finally, neohesperidin treatment phosphorylated AMPK, resulting in a rise of the HFD-downregulated proteins SIRT1 and PGC1α [211]. On its side, eriodyctiol has also demonstrated effects on diet-induced obesity. Diet-induced obese mice supplemented with eriodyctiol showed a reduction of hepatic TG, fatty acids and the size and number of lipid droplets accompanied with an increased fecal excretion of cholesterol and fatty acids [212]. It is worth to mention that eriodyctiol decreased the enzymatic activity of malic enzyme (ME), FASN, phosphatide phosphohydrolase (PAP) and downregulated the expression of *Srepb1c, Acc and Fasn* [212]. These data indicate that eriodyctiol improved the hepatic steatosis caused by a HFD by decreasing hepatic lipogenesis and increasing the hepatic FAO. On the other hand, alpinetin, an O-methylated flavanone, improved HFD-induced NAFLD via ameliorating oxidative stress, inflammatory response and lipid metabolism. Alpinetin decreased *Scd1, Fasn, Srebp1c, Lxrα, Elovl2* and *Irs1* expressions, and increased PPARα levels [213].

In humans a randomized placebo-controlled, double-blind clinical trial with NAFLD patients shown the effect of hesperidin supplementation [214]. Patients who follow healthy lifestyle habits and supplemented their diet with hesperidin have a significant reduction of ALT, glutamyl-transferase, total cholesterol, hepatic steatosis, C reactive protein and TNFα, proving the scope of hesperidin [214]. One of the possible mechanisms underlying the effects of flavanones on metabolism goes through the FGF21 and AMPK/Sirt1/PGC1α signaling axis.

### 5.2. Flavanones Induce Browning in Adipose Tissue

As other flavonoids, flavanones can also modulate lipid metabolism in adipose tissue as well as induce browning in WAT, and activate in BAT [166] as well as reduce the characteristic obese-macrophage infiltration in adipose tissue [215].

In HFD fed mice, hesperetin supplementation on its side showed metabolic health effects in adipose tissue, concretely is able to reduce mesenteric adipose weight and decrease leptin levels [216]. In this case, lipid metabolism was not changed nor in liver nor in WAT. On the other hand, a characteristic of obesity is the recruitment of immune cells by adipose tissue that leads to metabolic disorders such as insulin resistance. In a short-term HFD mice model, naringenin can suppress neutrophil and macrophage infiltration into adipose tissue [215]. Concretely it can inhibit the expression of several chemokines like MCP-1 and MCP-3 [217]. Eriodyctiol (ED) supplementation on its side lowered the adiposity in diet-induced obese mice by regulating gene expression. ED-supplemented mice showed reduced weight of all the WAT depots but also a downregulated expression of adipocyte genes involved in lipid uptake (*Cd36*, and *Lpl*) and lipogenesis (*Srebp1, Acc, and Scd1*), an upregulation of the *Ucp1*, with no changes in FAO genes such as *Adrb3, Cpt2, Pgc1α, Pgc1β,* and *Cox8b* genes [212].

Another beneficial effect of flavanones in adipose tissue is related to EE and thermogenesis. It has been demonstrated that in human white adipocytes and in scWAT a treatment with naringenin increased the expression of genes associated with thermogenesis and FAO, including *Atgl* and *Ucp1* as well as *Pgc1*α and *Pgc1*β that can mediate the PPARδ-dependent transcriptional responses involved in mitochondrial biogenesis and uncoupling phenotype. Moreover, naringenin administration increased the expression of insulin sensitivity-related proteins such as *Glut4*, *adiponectin*, and *Chrebp* [218]. These data indicate that naringenin may promote the conversion of human WAT to a brown/beige adipose tissue. Similarly, in HFD-obese mouse model, the induction of brown-like adipocyte formation on WAT was described by supplementing the diet with a flavanones-rich extract from *Citrus reticulata* [219]. The main phytochemical components of a water extraction of *Citrus reticulata* in were synephrine, narirutin, hesperidin, nobiletin, and tangeretin. Among flavanones, citrus also contain synephrine that is an alkaloid which binds to β_3_AR in adipose tissue promoting lipolysis and thermogenesis [220]. Dietary supplementation with this citrus extract reduced body weight gain, epididymal fat weight, fasting blood glucose, serum levels of TG and total cholesterol, and lipid accumulation in liver and WAT as well as activated FAO and induced the browning phenotype [219]. These animals showed increased levels of *Ucp1* in the iWAT and an upregulation of *Prdm16*, *transmembrane protein 26 (Tmem26), cluster of differentiation 137 (CD137),* and *Cidea* [219].

In the same way it has been published that hesperidin induced browning in retroperitoneal WAT (rWAT) but not in iWAT of Western diet-fed rats. Hesperidin decreased the size of adipocytes and induced the formation of multilocular and positive-UCP1 and CIDEA brown-like adipocytes. Besides the induction browning, hesperidin also enhanced the expression of *Ucp1* in BAT [221]. In contrast, it has been recently published a study where not hesperidin but its monoglycosyl has the capacity to induce brown-like adipocyte formation in HFD-fed mice [222]. In this case, α-monoglucosyl hesperidin increased EE and reduced body fat accumulation by stimulating the browning phenotype in the iWAT. iWAT adipocytes of supplemented mice exhibited a multilocular phenotype and were UCP1-positive cells. The iWAT of these animals also showed increased levels of COXIV. No effects were observed in BAT nor in other WAT depots [222].

In a human randomized double-blind placebo-controlled trial with moderate high BMI subjects, it’s shown that glycosylated hesperidin decreased significantly abdominal and subcutaneous fat area when is supplemented with caffeine [223].

### 5.3. Flavanones Are Neuroprotective against Several CNS Injuries

There is low information about the effects of flavanones on CNS to combat obesity. It has been demonstrated that quercetin, naringenin and berberine can modulate glucose homeostasis in the brain of STZ-induced diabetic rats through the regulation of glucose transporters and other key components of insulin signaling pathway [224].

Most of the studies that show the neuroprotective role of flavanones have been performed using animal with CNS-induced injuries. In a rat model of global cerebral ischemia reperfusion (I/R), pinocembrin (a honey flavanone) exerted antioxidant, anti-inflammatory and anti-apoptotic effects. [225] as well as inhibited autophagy on the hippocampus [226]. Moreover, naringenin and eriodyctiol exert effects in ischemic stroke, promoting cortical cell proliferation, inhibiting apoptosis and reducing oxidative stress in rodent models [227,228]. In a similar way, the induction of neurotoxicity by lipopolysaccharide (LPS) administration in mice can be ameliorated by the coadministration of hesperetin or naringenin that reduced the expression of inflammatory cytokines, attenuated the generation of reactive oxygen species/lipid peroxidation and enhanced the antioxidant capacity in CNS [229,230]. Furthermore, hesperetin enhanced synaptic integrity, cognition and memory processes by increasing the levels p-CREB, postsynaptic density protein-95 (PSD-95) and syntaxin proteins [229] and naringenin decreased the acetylcholinesterase (AChe) activity [230]. Other mental stresses such as social defeat stress, depression and autistic-like behaviors can also be counteract with flavanones in rodent models [231,232,233]. Hesperidin and naringenin have demonstrated positive effects by increasing the resilience through a reduction in the levels of interleukins and corticosterone thus suppressing the chronic inflammation caused by kynurenine pathway related to depression [234] and inhibiting the AChe activity, the oxidative stress as well as neuroinflammation [235].

## 6. Flavonols

Flavonols are widely distributed in plants and are present as minor compound in many polyphenol-rich foods. Their synthesis is stimulated by light and they accumulate in the skin of fruits and vegetables being absent in the flesh. The main dietetic flavonols are quercetin, kaempferol, isorhamnetin, fisetin, and myricetin [48,236,237].

Quercetin is found in capers, lovage (*Levisticum officinale*) apples, seeds of tomatoes, berries, red onions, grapes, cherries, broccoli, pepper, coriander, citrus fruits, fennel, flowers, leaves pepper and teas (*Camellia sinensis*) and it is the skeleton of other flavonoids, such as hesperidin, naringenin, and rutin. Rutin, rutoside or sophorin are the glycosylated form of quercetin and can be extracted from buckwheat, oranges, grapes, lemons, limes, peaches, and berries [238]. Kaempferol is abundant in apples, grapes, onions, tomatoes, teas, potatoes, beans, broccoli, spinaches, and some edible berries. Isorhamnetin is commonly found in medicinal plants such as ginko (*Ginkgo biloba)*, sea-buckthorn *(Hippophae rhamnoides*) and *Oenanthe javanica.* Myricetin is found in teas, wines, berries, fruits and vegetables. Fisetin is abundant in apples, grapes, persimmon, cucumber, onions and strawberries. Finally, morin is present in *Prunus dulcis, Chlorophora tinctoria L*., and fruits such as guava and figs [45].

As other groups of flavonoids, flavonols have shown healthy effects. They exhibit anticarcinogenic, anti-inflammatory, and antioxidant activities but also anti-obesity and antidiabetic properties in animal models and in humans where flavonols consumption has been associated to a lower risk of type 2 diabetes [43,236,237,238,239,240,241,242,243]. Some flavonols inhibited carbohydrate absorption thus lowering postprandial blood glucose mainly through the inhibition of the α-glucosidase activity but also by inhibiting glucose transporters (GLUT2, SGLT1) or other enzymes such as maltase or saccharase [236]. Finally, a combination of quercetin and resveratrol have shown the capacity to reduce obesity in HFD-fed rats by modulating gut microbiota [244].

Due to the high number of publications and previous reviews [45,48,238], in the present work only the most recent data have been included.

### 6.1. Flavonols Exert Beneficial Effects on Lipid Steatosis by Regulating Lipid Metabolism, Inflammation and Oxidative Stress

Quercetin enhanced hepatic insulin sensitivity and reduced liver fat content and ameliorated hepatic steatosis [245]. Quercetin diminished the mRNA and protein levels of CD36 and MSR1, upregulated the levels of LC3II and downregulated p62 and mTOR thus suggesting an autophagy lysosomal degradation as the potential hepatoprotective mechanism of quercetin [245]. From another point of view the effects and mechanisms of quercetin against NAFLD were analyzed through a metabolomic approach [246]. Treatment with quercetin decreased AST and ALT levels in serum and reduced lipid droplets and hepatocyte swelling in rats fed a high fat/high sucrose diet. A metabolomic analysis indicated that quercetin modified fatty acid- inflammation- and oxidative stress-related metabolites among others. In this case, the effects of quercetin were more evident in 30-day NAFLD induction than in 50 days, thus indicating that dietary quercetin may be beneficial in early stages of NAFLD development [246]. Besides the effects of quercetin alone there are several studies where quercetin is used in combination with other compounds. The beneficial effects of quercetin in NAFLD development increased synergistically when quercetin is administered within benifuuki, a tea that contains EGCG. Both compounds administered to rats fed high fat/high cholesterol diet were more effective to downregulate *Fasn* and *Scd1* showing higher effects on their lipid-lowering effects alone [247]. In a similar way, the combination of quercetin with resveratrol ameliorated fatty liver in rats by improving the antioxidant capacity of the liver [248]. Finally, a combination of borage seed oil (as a source of linoleic (18:2n-6; LA) and gamma-linolenic (18:3n-6; GLA) acids and quercetin improved liver steatosis in obese rats [249].

On its side, isoquercetin (IQ), a glucoside derivative of quercetin has demonstrated beneficial effects in NAFLD by improving hepatic lipid accumulation via an AMPK dependent way in HFD-induced NAFLD rats [250]. Concretely, IQ treatment enhanced the phosphorylation of AMPK and ACC and reversed the downregulation of liver kinase β1 (LKβ1) and Calcium/calmodulin-dependent protein kinase kinase-1 (CaMKK1) caused by HFD. The activation of AMPK modulated the expression of lipogenic and lipolytic genes, such as *Fasn, Srebp1c, Pparγ and Cpt1a*. Moreover, IQ supplementation upregulated PPARα and downregulated nuclear factor-kB (NF-kB) protein levels [250].

As quercetin, kaempferol is also able to reduce lipid accumulation in liver of obese rodent models. In dyslipidemia-induced mice, kaempferol inhibited PKB (Akt) and SREBP-1 activities and blocked the Akt/mTOR pathway, thus inducing hepatic autophagy and decreasing hepatic lipid content [251]. Similarly, in ApoE deficient mice fed with a HFD, kaempferol attenuated metabolic syndrome via interacting with LXR receptors and inhibiting posttranslational activation of SREBP-1. Both effects contributed to the reduction of plasma and serum TG [252].

Other flavonols with positive effect in the liver are fisetin, dihydromyricetin or rutin. Obese rats fed with a high fat/high sucrose diet and supplemented with fisetin showed a decreased in body weight and hepatic lipid content as well as an improvement in the lipid profile (low levels of TG, total cholesterol, LDL) and liver functionality (reduced levels of ALT and AST). The hepatic nuclear receptor 4α (HNF4α) has been pointed out as the key factor in the hepatic effects of fisetin. Fisetin upregulated *Hnf4a* gene expression, increased nuclear lipin-1 levels. Moreover, fisetin promoted FAO, diminished FASN activity, enhanced hepatic antioxidant capacity and decreased the hepatic poly (ADP-ribose) polymerase 1 (PARP1) activity, a DNA repair enzyme, and thioredoxin-interacting protein (TXNIP) that is important for maintaining the redox status [253]. Through the regulation of SIRT3 signaling, dihydromyricetin have showed the ability to ameliorate NAFLD in HFD-fed mice. Dihydromyricetin increased *Sirt3* expression via activation of the AMPK/PGC1α/estrogen-related receptor α (ERRα) cascade thus improving mitochondrial capacity and restored redox homeostasis [254]. In a similar way, rutin lowered TG content and the abundance of lipid droplets in NAFLD-induced HFD fed mice. Rutin treatment restored the expression of *Pparα* and *Cpt1a* and *Cpt2*, while downregulated *Srebp-1c*, *diglyceride acyltransferase 1 and 2 (Dgat-1 and 2* and *Acc*. These effects enhanced FAO and diminished lipid synthesis. In addition, rutin repressed the autophagy in the liver [255]. On its side, the rutin derivate, troxerutin (TRX), has also demonstrated effectiveness against metabolic disorders in a rat model of hereditary hypertriglyceridemia (HHTg) non-obese model of MetS [256]. The treatment with TRX lowered the levels of hepatic cholesterol and reduced the expression of cholesterol and lipid synthesis genes (*Hydroxymethylglutaryl-CoA reductase* (*Hmgcr), Srebp2* and *Scd1*) as well as decreased lipoperoxidation and increased the activity of antioxidant enzymes [256]. Moreover, these animals exhibited higher levels of adiponectin in serum [256].

Besides the effects of flavonols by itself, favonols-rich extracts have also been tested in fatty liver-associated diseases. A *Sicyos angulatus* extract that contains kaempferol as the main flavonol administered to a HFD-induced obese mice lowered plasma levels of ALT and AST and the hepatic lipid content. The *Sicyos angulatus* extract impacted on lipid metabolism by repressing the expression of genes related to fatty acid and TG synthesis (*Acc1, Fasn Scd1* and *Dgat*) and of the key transcription factors that regulate lipogenesis (*Srebp-1c* and *Pparγ*) [257]. Another source of kaempferol, quercetin and derivates is Sanglan Tea (SLT), a Chinese medicine-based formulation consumed for the effective management of obesity-associated complications. It has been demonstrated that dietary SLT supplementation prevented body weight gain and fatty liver and ameliorated insulin resistance in HFD-induced obese mice. SLT improved the serum lipid profile (lower levels of TG, Total cholesterol and LDL) and reduced the ALT and AST circulating levels. The liver of these animals displayed less lipid droplets and a downregulation of the lipogenic genes *(Lxrα, Fasn, Acacb, Srebf-1,* and *Scd1)* and the adipogenesis-related genes (*Pparγ, C/ebpα* and *Ap2*) that are induced under HFD [258].

In a similar way, the flower of *Prunus persica* commonly known as peach blossom has demonstrated that capacity to reduce body weight, abdominal fat mass, serum glucose, ALT, AST, and liver and spleen weights compared to a HFD fed mice. This flower is rich in flavonoids and phenolic phytochemicals with chlorogenic acid, kaempferol, quercetin and its derivatives as its major compounds. The supplementation with this flower suppressed hepatic expression of lipogenic genes (*Scd1, Scd2, Fasn*) and increased the mRNA levels of FAO genes (*Cpt1a*), thus modifying he lipid metabolism in HFD-fed mice [259]. Furthermore, a mulberry leaf powder also showed effects on liver gene expression in a mice model of hepatic steatosis induced by a western diet. Liver weight, plasma TG and liver enzymes ALT and AST were reduced in treated-animals. A global hepatic gene expression analysis revealed that supplemented mice displayed a downregulation in inflammation-related genes and an upregulation in liver regeneration-related genes [260]. Finally, a 70% ethanol extract from leaves of *Moringa oleifera (MO)* that contains different flavonols and flavones such as quercetin and kaempferol and their derivates. reduced glucose and insulin but also the total cholesterol, TG and LDL serum and increased the HDL in high-fat diet obese rats as well as downregulated hepatic expression of *Fasn* and *Hmgcr* [261].

Through a network pharmacological approach Nie et al. [262] highlighted that Chaihu shugan powder (CSP) may exert its beneficial effects against NAFLD through the interaction of its main compounds with nuclear receptors. Through a molecular docking approach, they screened PPARγ, FXR, PPARα, RARα and PPARδ and quercetin, kaempferol, naringenin, isorhamnetin and nobiletin interactions. To confirm the results of docking, an in vivo approach was done using NAFLD-induced rats. The NAFLD-induced rats treated with CSP exhibited ameliorated effects in body weight, hepatic histopathology and serum and liver lipids. Moreover, the mRNA levels of *Pparγ, FXR, Pparα* and *Rarα* were modified suggesting nuclear receptors regulation as a potential molecular mechanism underlying the effects of CSP [262].

Adiponectin signaling and AMPK activation have been also pointed out as possible mechanisms underlying the effects of flavonols in the liver. An extract of black soybean leaves (EBL), which mainly contains quercetin glycosides and isorhamnetin glycosides was administered to HFD-fed mice. EBL supplementation reduced body weight, fasting glucose, TG, total cholesterol and non-esterified fatty acid levels as well as hepatic steatosis. EBL supplementation increased the levels of adiponectin and the expression of adiponectin-receptors in the liver (AdipoR1 and AdipoR2) thus restoring adiponectin signaling pathway [263]. Downstream of the adiponectin signaling there is the activation of AMPK and FAO, the suppression of fatty acid synthesis and the improvement of insulin signaling [264]. Moreover, the mRNA levels of *Pgc1, Pparα, Pparδ, Pparγ, Acc, Fasn, Cpt1a, Glut2, FoxO1 and Irs1* were partially or totally normalized in HFD-EBL-supplemented animals [263].

Finally, it has been described that part of the mechanisms involving the hepatic beneficial effects of flavonols may be mediated by gut microbiota. An experimental approach of gut microbiota transplantation revealed a gut–liver axis where the *Akkermansia* genus have a key role on the quercetin protecting effects against obesity-associated NAFLD development. [247]. In a similar way, kaempferol blunted part of the effects of HFD in gut microbiota diversity. HFD fed mice displayed a reduced microbial diversity that it is mostly reversed by kaempferol [265]. Furthermore, IQ combined with inulin attenuated weight gain, improved glucose tolerance and insulin sensitivity and reduced lipid accumulation in the liver, adipocyte hypertrophy in WAT and diminished the circulating levels of leptin in HFD-fed mice probably through the modulation of gut microbiota [266].

### 6.2. Flavonols Impact on WAT Where They Modulate Lipid Metabolism and Induce Browning

Several studies with animal models showed that flavonols can protect mice or rats from HFD obesity by reducing body weight gain and lipid accumulation in WAT via reducing inflammation, modifying lipid metabolism, increasing EE, inducing browning of WAT and activating BAT [174,242,267,268,269].

Quercetin and quercetin-rich red onion (ROE) ameliorated diet-induced WAT expansion and inflammation in HFD-fed mice [270]. Quercetin and ROE ameliorated adipocyte size and number compared to HFD fed mice in WAT depots and induced a multilocular phenotype typical of BAT [270]. Moreover, quercetin and ROE diminished the HFD-increased levels of leptin. Besides its impact on adipose tissue phenotype, quercetin and ROE supplementation also attenuated the inflammatory profile induced by HFD in WAT [270]. Similarly, a quercetin-rich supplement administered to diet-induced obese rats decreased body fat and adipocyte size of the perirenal WAT as well as increased adiponectin circulating levels [271]. Quercetin-rich supplement attenuated the upregulation of genes related to lipid synthesis such as *Acc, Fasn*, *HMG-CoA reductase*, *Lpl, Ap2,* and *Fatty acid transporter protein 1 (Fatp1)* caused by HFD; and upregulated the HFD-downregulated genes such as *Atgl, Hsl, Ampk, Acox, Pparα,* and *Cpt1a* [271]. In diet-induced obese mice quercetin administration decreased plasma TG levels without affecting food intake, body composition, or EE [272]. Quercetin enhanced the uptake of [^3^H]-oleate derived from labeled lipoprotein-like particles in the scWAT [272]. On the other side Perdicaro et al. demonstrated that quercetin attenuated adipose tissue hypertrophy, reduced the adipocyte size but activated the adipogenesis in HFD-fed rats. Quercetin supplemented rats showed increased levels of angiogenic (*Vascular endothelial growth factor 1* and *2 (Vegf1, Vegf2*) and adipogenic (*Pparg* and *C/ebpa*) markers but also mitigated inflammation, and reticulum stress [273].

Together with their capacity to modulate lipid metabolism, flavonols are also able to induce browning in WAT depots. Quercetin treatment increased the expression of *Ucp1, Pgc1α* and *Elovl3* in WAT [272,274]. In a similar way, the administration of onion peel extract (rich in quercetin) to HFD-fed mice upregulated markers of BAT (*Prdm16, Pgc1α, Ucp1, Fgf21, Cidea*) in perirenal and scWAT [275]. It has been described that the induction of browning was mediated at least in part through the activation of the AMPK and the SIRT1 or via sympathetic stimulation. The quercetin-supplemented HFD-fed mice displayed higher levels of plasma norepinephrine and of PKA protein levels in scWAT [274]. Besides the activation of PKA signaling, it has been described that quercetin also increased SIRT1 protein levels and pAMPK in visceral WAT [276]. Although most of the studies showed positive effects of quercetin, this flavonol did not induce significant effects on the adipose tissue weights of rats fed an obesogenic diet except when combined with resveratrol (RSV). The treatment with quercetin and RSV but not with just quercetin or RSV promoted multilocular UCP1-positive adipocytes that also displayed increased levels of browning markers (*Cidea, bone morphogenic protein 4 (Bmp4), Homeobox C9 (Hoxc9), Solute Carrier Family 27 Member 1 (Slc27a1), Tmem26* and *proton/amino acid symporter (Pat2))* and genes related to catabolic pathways (*Atgl* and *ATP synthase subunit delta (Atp5d))* in perirenal WAT. Regarding BAT, the supplementation with RSV and quercetin upregulated *Cidea* and *Ucp1* expression, thus indicating more thermogenic capacity in this tissue [277].

It is worth to mention that quercetin effectiveness is specie dependent. Studies in rats usually showed more effects than in mice whilst in humans the results are still unclear. In rodent models the levels of quercetin reached after its administration are higher than in humans [269]. Similar to quercetin, isoquercetin (IQ), a quercetin glycoside with greater bioavailability than quercetin, also exerts positive effects in WAT. In normal diet-fed mice IQ supplementation decreased WAT weight and increased pAMPK levels in WAT as well as in liver and muscle. Moreover, IQ reduced the expression of *Pparγ, C/ebpα, C/ebpβ* and *Srebp1* whilst increased the expression of *Ucp2, Pgc1α, Prdm16, Sirt1* and *Cpt1a* in WAT, suggesting less adipogenesis, enhanced FAO and browning [278].

On its side, rutin administration to db/db mice and diet-induced mice reduced body weight gain and improved adiposity (smaller lipid droplets) mainly by increasing EE [279]. These animals exhibited higher core temperature when submitted to a cold environment indicating enhanced BAT activity. Rutin-treated animals overexpressed BAT markers (*Ucp1, Cidea, Prdm16*), FAO-related genes (*Cpt1a, Mcad, Ppar*α *and Pgc1α*), mitochondrial biogenic transcription factors (*tfam, Nrf1, Nrf2)* and more copies of mitochondrial DNA in BAT [279]. Besides BAT, rutin also affected scWAT, where induces browning (upregulation of BAT-specific genes, including *Ucp1, Pgc1α, Pgc1β, Cpt1a, Pparα, Tfam, Nrf1* and *Nrf2*...) [279]. The molecular mechanism underlying these effects may go through the Sirt1 activation. It has been demonstrated that rutin was able to directly bind to Sirt1 protein and activate the SIRT/PGC1*α*/NRF2/Tfam signaling pathway [279]. On the other hand, rutin combined with exercise (treadmill running) in diet-induced obese mice increased the mRNA levels of *adiponectin*, the protein levels of PPARγ, the binding immunoglobulin protein (BIP), and the phosphorylated form of c-Jun terminal quinase (JNK) and reduced disulfide-bond A oxidoreductase-like protein (DsbA-L). These profile indicated an improvement on the ER stress and on adipose tissue functionality [280].

When instead of flavonols, plant extracts were used similar effects were observed. A 70% ethanol extract of *Moringa oleifera (MO)* that mainly contains quercetin, kaempferol and their derivates induced the expression of *Glut4*, *adiponectin, omentin* and upregulated *Pparα* and *melanocortin-4 receptor (MC4R)* on the WAT of diet-induced obese rats. [261]. *Cuscuta pedicellata* and some of its isolated compounds, including kaempferol, quercetin and some derivates were suggested to have an anti-obesity effect in HFD-fed rats. Supplemented animals showed a reduction in HOMA-IR and oxidative stress as well as exhibited an upregulation of *Ucp1* and *Cpt1a* expression in BAT [281]. Finally, through a high-throughput metabolomic approach it has been described that the consumption of a hawthorn ethanol extract that contains chlorogenic acid, hyperoside, isoquercetin, rutin, vitexin, quercetin, and apigenin affected several metabolic pathways including: fatty acid biosynthesis, galactose metabolism, biosynthesis of unsaturated fatty acids, arginine and proline metabolism, alanine, aspartate and glutamate metabolism, glycerolipid metabolism and steroid biosynthesis [282].

### 6.3. Flavonols: Neuroprotection in Neurodegenerative Diseases

Flavonols have shown neuroprotective effects in neurodegenerative diseases. Quercetin, rutin and some other flavonols have exhibited positive effects against pathologies such as Alzheimer’s Disease (AD), Parkinson’s disease, Huntington’s Disease, multiple sclerosis, brain ischemic injury, epilepsy neurotoxins but also for aging cognitive alterations [238,283,284,285,286,287,288]. Furthermore, flavonols have also demonstrated beneficial effects in the CNS alterations caused by HFD.

It is well-known that HFD induces oxidative stress in brain that may lead to neurodegenerative diseases. In HFD-fed mice, quercetin ameliorated the cognitive and memory impairment and enhanced the expression of *phosphatidylinositol-4,5-bisphosphate 3-kinase (PI3K), PKB/Akt, Creb,* and *brain-derived neurotrophic factor (Bdnf)* [289]. In a similar way, in HFD-fed mice, *Acer okamotoanum* and its main bioactive compound isoquercitin improved cognitive function by inhibiting the ROS production, the lipid peroxidation and nitric oxide formation, thus reducing oxidative stress [290]. Furthermore, it has been described that obesity induces hypothalamic inflammation and activates microglia. In diet-induced obese mice, quercetin supplementation reduced the levels of inflammatory cytokines and microglia activation markers in the hypothalamus [291]. Quercetin has also showed positive effects in streptozotocin (STZ)-induced AD rats where improved memory impairment and the anxiogenic-like behavior induced by STZ. In these rats, quercetin prevented the acetylcholinesterase (AChE) overactivity and the increased malondialdehyde levels caused by STZ [292]. Finally, quercetin showed capacity to modulate several kinases signaling cascades involved in synaptic plasticity such as the PI3K/Akt, protein kinase C (PKC) and mitogen-activated protein kinase (MAPK) [293].

## 7. Isoflavones

Isoflavones, also known as phytoestrogens, are flavonoids with a limited distribution in plant kingdom. They are found in leguminous plants such as soybean, kudzu, red clover, fava beans, alfalfa, chickpeas or peanuts but also soy-based foods (tofu, soymilk, miso…) and some pants such the *Puerariae* genus [42,294]. Genistein and daidzein are the most representative dietary isoflavones.

Although there are several human clinical studies studying soy isoflavone consumption and diabetes the data obtained are not conclusive. Some evidence suggests that long-term intake of isoflavones may improve insulin resistance in type 2 diabetic patients and have anti-obesity effects [295,296,297,298,299]. In animal studies, isoflavones have showed antidiabetic and anti-obesity activities [45,236,297,300]. The beneficial effects of isoflavones include the improvement of insulin sensitivity, lipid profile and adiposity [45,49,301,302,303].

### 7.1. Isoflavones Reduced H Steatosis by Modulating Lipid Metabolism

Like many of the other flavonoids, isoflavones also exert an hepatoprotective action [49]. A recent publication using data of the National Health and Nutrition Examination Survey from 1999 to 2010 in the USA describes an inverse correlation between urinary genistein levels and serum ALT levels in males but not in females [304]. On the other hand, in NAFLD-rodent models, genistein supplementation decreased fat accumulation, inflammation, hepatic steatosis and liver fibrosis in animal models and in humans [302]. These effects on hepatic steatosis have been described both in short- and long-term interventions [305].

One of the mechanisms proposed is the blockade of aldose reductase (AR)/polyol pathway. It has been described that some isoflavones are AR inhibitors. The inhibition of the AR/polyol pathway reduces fructose production and hepatic fat accumulation in high glucose diets as well as improved PPARα activity and enhanced FAO, thus attenuating liver steatosis in HFD-obese models [306]. Moreover, the blockade of AR/polyol pathway reduced the CYP2E1-mediated oxidative stress [306]. Other mechanism suggested for isoflavones is the downregulation of PPARγ and fat-specific protein 27 (FSP27) together with a reduction of fatty acid synthesis and increased lipolysis [307]. This mechanism was described in female rats fed with a 20% casein-diet and supplemented with soy isoflavones [307].

Effects via the activation of AMPK has been also described for genistein [308,309]. Hepatic activation of AMPK drives to an inhibition of cholesterol and fatty acid synthesis and an enhancement of FAO [310]. In high fat/high sucrose-fed rats, genistein improved lipid metabolism and ameliorated hepatic lipid accumulation. P-AMPK and p-ACC were increased while SREBP1 protein levels were decreased. Moreover, genistein downregulated the expression of *Fasn, glycerol-3-phosphate acyltransferase (Gpat)* as well as upregulated *Pparα, Cpt1a* and *Acox* [309]. A similar effect on NAFLD has been described with Puerarin, a major bioactive isoflavone compound isolated from the roots of the *Pueraria lobata.* Puerarin attenuated NAFLD development in high fat/high sucrose-fed mice via the activation of the Poly(ADP-ribose) polymerase 1 (PARP-1)/PI3K/Akt signaling pathway and lately the improvement of the mitochondrial function [311]. In HFD-obese mice, puerarin reduced TG, total cholesterol and leptin serum levels as well as decreased the hepatic lipid content. Puerarin inactivated FASN and activated AMPK, CPT and HSL as well as increased the protein levels of PPARγ. These data indicated that puerarin regulated lipid metabolism by reducing lipid synthesis and enhancing lipid consumption [312].

Positive effects on NAFLD has been also observed by combining soluble soybean polysaccharides and genistein. This combination increased the bioavailability of genistein and administered to HFD-fed mice prevented weight gain, oxidative stress inflammation and dyslipidemia. These effects on lipid profile have been related to an activation of AMPK and PPARα/PPARγ pathways and changes in the mRNA levels of *Fasn, Acc, Srebp1c* and *adipose differentiation-related protein (Adrp)* [313].

Besides genistein some of its derivatives are also active. Sophoricoside, a genistein derivate isolated from the *Sophora japonica* L, has been tested in high fructose-fed mice. Administration of sophoricoside diminished body and liver weight as well as reduced hepatic cholesterol and TG and serum levels of ALT, AST and LDL whilst increased the levels of circulating HDL. Moreover, the livers of treated-mice displayed a better inflammatory profile and an increased antioxidant capacity [314]. Calycosin, an o-methylated isoflavone showed positive effects against NAFLD-induced in HFD-fed mice. Calycosin improved insulin sensitivity, decreased the levels of ALT and AST and increased the levels of adiponectin. In the liver, calycosin blocked gluconeogenesis and lipogenesis by suppressing PEPCK G6Pase, SREBP1c and FASN, as well as induced the expression of *Gsk3β, Glut4,* increased the phosphorylation of Irs1 and Irs2 and activated farnesoid X receptor (FXR) [315].

Similar to isolated compounds, soy isoflavones (that includes genistein, daidzein and glycitein) or a soy protein preparation also reverted hepatic steatosis when administered to obese female Zucker or HFD-obese rats. Soy isoflavones reduced hepatic lipid accumulation, improved serum levels of ALT and downregulated *Srebp1c* and *Fasn* levels as well as increased the protein levels of PPARα indicating less lipogenesis and more FAO [316]. In a similar way, the intake of soy protein with isoflavones decreased the liver steatosis, reduced the levels of AST and ALT and increased the levels of leptin in female Zucker obese rats [305].

Apart from the effects of isoflavones on lipid metabolism they also exhibit anti-inflammatory properties. Genistein protected against NAFLD by targeting the arachidonic acid cascade that is responsible for the chronic inflammation [317]. Genistein supplementation to HFD-fed mice blocked the synthesis of ciclooxigeanse-1 activity and thromboxane A2 [317]. Other mechanism to explain the anti-inflammatory effect of genistein is the promotion of miR-451 [318]. In humans a randomized controlled trial described that genistein supplementation improved the inflammatory state in NAFLD patients [319].

### 7.2. Isoflavones Ameliorate the Weight Gain in Diet-Induced Obesity Models and Improve Lipid Metabolism in Adipose Tissue

It has been widely described that isoflavones are able to control food satiety and appetite, to ameliorate the body weight gain and fat accumulation in rodent models of obesity, to modulate fatty acid metabolism and to induce browning and BAT activation which make its use in nutritional interventions as a promising approach for weight management therapies [269]. Isoflavones reach and affect adipose tissue as it was demonstrated through a whole-transcriptome microarray analysis of the perigonadal WAT from mice fed either control diet or a soybean extract diet containing a genistein/daidzein mix. This study described the impact of soy isoflavones on adipose tissue describing 437 downregulated genes and 546 upregulated [320].

In HFD-fed rats, soy isoflavones attenuated diet-induced obesity mainly by reducing the visceral WAT depot (lower hypertrophy and less lipid accumulation). Soy isoflavones supplementation downregulated fat synthesis (reduced SREBP1 protein levels) and upregulated lipolysis (increased ATGL protein levels) in visceral WAT via the activation of AMPK and the inhibition of SREBP1 [321]. In a similar way, 6,8-diprenylgenistein (DPG), a major isoflavone of *Cudrania tricuspidata* fruits decreased the body weight of HFD-induced obese mice at least in part by the suppression of de novo lipogenesis via the AMPK activation [322]. This isoflavone reduced the expression of lipogenic genes by regulating Pparγ and C/EBPα transcriptional activity as well as leptin and adiponectin levels. DPG also regulated ACC and HMGCR [322].

Isoflavones are also present in fermented soy products. The heathy properties of these products have been also evaluated. Fermented soybean meal (SBM) administered to HFD-fed rats showed positive effects on the obese profile of these animals. The body weight gain, as well as weights of abdominal and epididymal fat were reduced. Also, the lipid profile was improved. Supplemented rats exhibited lower levels of TG, total cholesterol and LDL and higher levels of HDL compared to HFD-non supplemented rats. Moreover, in WAT, there were a decrease on the hepatic lipogenesis (downregulation of *Fasn* and *Acc)* and an increase on lipolysis (upregulation of *Lpl*) [323].

Besides their effects on lipid metabolism, isoflavones also induce browning and BAT activation [166]. Genistein administration to HFD-fed mice reduced body weight gain and scWAT mass and induced the expression of *Ucp1* and *Cidea* in WAT, indicating a browning phenotype [324]. Genistein may induce the browning phenotype by a direct upregulation of *Ucp1* expression or through an indirect pathway that would imply irisin signaling. Irisin is a myokine that induces the expression of *Ucp1* and *Tmem26* in preadipocytes [325]. This indirect mechanism describes an induction of the PGC-1α/FNDC5 pathway in skeletal muscle that lead to an increase of irisin production and secretion [325].

Formononetin and puerarin also modulate adipogenesis and thermogenesis. Formononetin attenuated visceral fat accumulation and increased EE in HFD-fed mice [326,327]. In vitro, this isoflavone downregulated P*parγ, C/ebpα* and *Srebp1* probably via AMPK/β-catenin signal transduction pathway that drove its antiadipogenic effect [326]. Moreover, formononetin induced *Ucp1* expression in primary culture of mouse adipocytes [327]. In a similar way, *Puerariae lobata* root extracts (PLR) activated browning in iWAT and regulated BAT activity [328]. PLR treatment caused weight loss and improved glucose metabolism in diet-induced obese mice as well as increased EE. In BAT, PLR upregulated *Ucp1* expression (but no other thermogenic markers) and in iWAT induced the expression of BAT markers (*Ucp1, Pparγ1, Pparγ2* and *Pparα),* thus indicating a brown-like phenotype [328].

Several studies focused on describing the mechanisms underlying the isoflavones’ effects have been performed in ovariectomized mice or rats. These models mimic menopausal stage in humans and are useful to analyze the potential role of isoflavones to counteract the increase of the adipose tissue that takes place during this period of life. In these rodent models, isoflavones exert positive effects on body weight gain and food intake as well as in fat pats enlargement [297]. In HFD-fed ovariectomized rats the administration of genistein decreased the body weight gain, improved insulin sensitivity and reduced plasma TG and cholesterol [329]. In liver, genistein blocked the lipogenic pathway by inhibiting p-ACC, SREBP-1, FASN and CD36 proteins. In retroperitoneal WAT, genistein diminished adiposity and adipocyte hypertrophy, inflammatory phenotype and induced browning. In iWAT, genistein-supplemented rats exhibited higher levels of UCP1, PRDM16, PGC-1α and CIDEA proteins and *Ppargc1a* and *Ucp-1* mRNAs [329]. Furthermore, isoflavones supplementation can modulate the metabolic effects of estradiol treatments in ovariectomized rats [330]. Finally, calycosin has demonstrated positive effects perivascular adipose tissue of obese mice. Through the adiponectin/AMPK/ endothelial nitric oxide synthase (eNOS) pathway, calycosin is able to restore at least in part the perivascular adipose tissue functionality [331].

### 7.3. Isoflavones Have Become Engaging Flavonoids in Neuronal Diseases due to Their Estrogenic-Like Structure and Its High Antioxidant Capacity

Obesity is a risk factor for neurodegenerative diseases essentially because it causes the neuroinflammation and oxidative stress. Isoflavones can ameliorate part of these effects as well as affect food intake and feeding behavior.

It has been described that daidzein administered to HFD-fed rats reduced food intake and attenuated body weight gain as well as improved glucose tolerance, adiponectin and leptin levels and increased the 17b-estradiol. In rat hippocampus, daidzein enhanced cell proliferation and reduced apoptosis and gliosis, thus exerting a neuroprotective effect against the brain injuries caused by diet [332]. On the other side, doenjang, a Korean traditional fermented soybean pastry alleviated hippocampal neuronal loss and enhanced cell proliferation in HFD-fed mice as well as reduced oxidative stress markers (less oxidative metabolites and lower levels of oxidative stress- and neuroinflammation-related genes). Dietary doenjang reduced Aβ and tau phosphorylation [333]. Furthermore, genistein has shown the capacity to improve metabolism and induce browning via hypothalamus gene expression regulation. Through a transcriptome analysis it was identified that the hypothalamic expression of *urocortin 3 (Ucn3), decidual protein induced by progesterone (Depp*), and *stanniocalcin1 (Stc1)* correlated with the browning markers in WAT and with insulin sensitivity [324].

Regarding neurodegenerative diseases isoflavones have shown protective properties. An extract of soybean isoflavone reduced the elevated oxidative stress parameters and reversed the overproduction of Aβ in rats with colchicine-induced neuronal damage [334]. In the same way, daidzein alone or mixed with genistein and glycitin isoflavones could reverse the cognitive impairments produced by scopolamine injection by activating the cholinergic system and the BDNF/ERK/CREB signaling pathway in mice [335,336], thus reinforcing the idea that soy isoflavones may be a good candidate for the treatment of neurodegenerative diseases. Besides the BDNF/ERK/CREB signaling pathway, it has been postulated that the Nrf2 signaling pathway can also be underlying the neuroprotective effects of isoflavones [337].

## 8. Flavones

Flavones is one of the largest groups of flavonoids with a high degree of chemical diversity. Some of the richest sources of flavones are parsley, celery, peppermint, and sage, which predominantly contain apigenin and luteolin as well as maize and citrus fruits. In general, flavones are found as glucosides in citrus fruits, vegetables, herbs and grains and although they represent a small fraction of the total flavonoid intake, they have shown health effects and anti-obesity properties [338,339]. As it is going to described latter, most of the studies that investigate the beneficial effects of flavones use them as aglycone and a scarce number of approaches deepen on the effects of flavones when consumed within the whole food and a feasible doses or in combination with other bioactive compounds.

### 8.1. Flavones Improved Liver Steatosis and Hepatic Inflammation

Flavones such as apigenin, luteolin, baicalin, vitexin, nobiletin among others prevented NAFLD and hepatic steatosis mainly by modulating lipid metabolism (increasing FAO and decreasing lipogenesis) and reducing oxidative stress and inflammation [340,341,342,343,344,345].

As many other flavonoids, some flavones also exert their hepatic effects by activating the AMPK enzyme. Vitexin, an apigenin flavone glucoside, for instance, when administered to HFD-fed mice reduced body and liver weight, triglyceride and cholesterol content in serum and liver and circulating levels of ALT and AST. Moreover, vitexin regulated lipid metabolism suppressing de novo lipogenesis by downregulating the expression of *Pparγ, C/ebpα, Srebp1c, Fasn,* and *Acc* and enhancing FAO and lipolysis by increasing the expression of *Pparα, Cpt1a* and *Atgl*) in an AMPK-dependent way that has been suggested may be activated by the binding of vitexin to the Leptin receptor [345].

In a similar way, luteolin, the principal yellow dye compound from *Reseda luteola*, or luteolin-enriched artichoke leaf extract alleviated hepatic alterations caused by a HFD by exerting anti-inflammatory activities and modulating lipid metabolism. Luteolin treatment of HFD-fed mice reduced hepatic lipotoxicity by improving the inflammatory profile, decreasing the extracellular matrix, enhancing the antioxidant capacity of the liver and increasing the FFA flux between liver and WAT [346]. A crosstalk between adipose tissue and liver has been suggested to explain the effects of luteolin on hepatic steatosis [347]. Moreover, luteolin and luteolin-enriched artichoke leaf extract administered to HFD-fed mice prevented hepatic steatosis (less and smaller lipid droplets, lower levels of C*idea*) and insulin resistance by suppressing lipogenesis and gluconeogenesis (suppression of PEPCK and G6Pase activities) and increasing FAO (more CPT1a activity and higher expression of *Pparα, Pgc1α and Pgc1β)* [342]. The repression of hepatocyte nuclear factor 4a and of LXR/SREBP1c signaling pathway has been described as putative molecular mechanisms for luteolin improvement of liver steatosis and NAFLD [348,349].

Regarding the capacity of flavones to modulate FAO, it has been described through a quantitative proteomic study that baicalin may act as an allosteric activator of CPT1a enzyme thus increasing the FA entrance to the mitochondria to undergo the β-oxidation in the liver [343]. Moreover, baicalin attenuated liver alterations by regulating the AMPK/ACC pathway in diet-induced obese mice [350]. Finally, baicalin is also a potent anti-inflammatory and antioxidant compound in a way that as other flavones also implied the nuclear erythroid 2-related factor 2 (Nrf2) activity in a cholestatic mice model [351].

It has been described that some flavones exert their hepatoprotective effects via the activation of the Nrf2 transcription factor. Nrf2 is a positive regulator of the expression of genes involved in the protection against oxidative stress as well as a negative regulator of genes that promote hepatic steatosis [352,353]. In this context, apigenin and scutellarin exerted their hepatoprotective activity via the activation of Nrf2. Scutellarin is a natural compound of *Erigeron breviscapus* (vant.) that in a HFD-fed mice attenuated obesity. It repressed lipogenesis and promoted FAO and cholesterol output besides its anti-inflammatory activity [340]. Moreover it has been described that scutellarin increased mRNA and/or protein levels of PPARγ, PGC1α, Nrf2, haem oxygenase-1 (HO-1), glutathione S-transferase (GST), NAD(P)H quinone dehydrogenase 1 (NQO1) and PI3K and AKT, whilst reduced nuclear factor kappa B (NF-κB), Kelch-like ECH-associated protein 1 (Keap1) [354,355]. By contrast, apigenin administration to HFD-fed mice inhibited the expression of PPARγ target genes via the translocation to the nucleus and activation of the Nrf2 transcription factor that seems to block PPARγ activity. Apigenin treatment downregulated the expression of genes related to lipid droplet formation (*Cidea, Plin2, fat storage inducing transmembrane protein 1 and 2 and)* as well as genes involved in FA uptake (*Fabp1* and *Lpl*), FAO (*Cpt1a, Pdk4, Acox1, Acaa2*) and lipogenesis *(Fasn, Scd11, Acaca)* [341]. On the other side, apigenin may act as a PPARγ modulator in a mouse model of obesity where it activated the p65/PPARγ complex translocation into the nucleus, thereby decreasing the NF-κB activation and favoring the M2 macrophage polarization [356] or blocking NLRP3 inflammasome assembly and the ROS production [357]. The capacity of flavones to modulate PPARγ activity and induce macrophage polarization to M2 phenotype has also been described for Chrysin in a HFD-fed mice model [358].

Finally, wogonin have shown beneficial effects on the liver steatosis development in a mice NAFLD model [359]. Concretely wogonin administration to HFD fed mice ameliorated the NAFLD progression via enhancing the PPARα/Adiponectin receptor R2 (AdipoR2) pathway. Wogonin induced the hepatic activity of PPARα and upregulated the levels of the AdipoR2. Moreover, wogonin also reduced the inflammatory profile and alleviated the hepatic oxidative stress [359].

Besides their effects alone, the combination of flavones with other bioactive compounds or polyphenols-rich extracts have also shown positive effects against hepatic steatosis [360].

### 8.2. Flavones Improved the Adipose Tissue Inflammation and Reduced the Macrophages Infiltration as Well as Enhanced the Thermogenic Capacity

Although flavones have been widely studied for their antioxidant and anti-inflammatory properties [338] their capacity to impact on adipose tissue metabolism and functionality cannot be underestimated.

Besides its reduction of the inflammatory phenotype in adipose tissue, apigenin administration to diet-induced obese mice ameliorated the body weight increment, reduced the visceral adiposity by inhibiting the adipogenesis via a STAT3/CD36 signaling pathway [361], decreased leptin and increased adiponectin [362] and induced energy expenditure mainly by promoting lipolysis and FAO as well as browning of WAT [363]. In scWAT, apigenin-treated mice exhibited a downregulation of adipogenic genes (*Pparγ, Lpl* and *aP2)* and of genes involved in lipogenesis (*Fasn* and *Scd1*) and a promotion of lipolysis by increasing the mRNA levels of *Atgl, Hsl, Forkhead box protein O1 (FoxO1) and Sirt1.* In BAT there is an increment of the p-AMPK and p-ACC levels, thus indicating that FAO is enhanced in this fat depot after apigenin administration. Finally, apigenin activated the thermogenesis in BAT (upregulation of *Ucp1* and *Pgc1α*) and induced the browning phenotype in scWAT (upregulation of *Ucp1*, *Pgc1α, Tmem26, Cited1*) [363]. Similar results were obtained with vitexin. Vitexin administration reduced the adipocyte size of HFD-fed mice and increased the p-AMPK levels in eWAT followed by a downregulation of C/EBPa and FASN protein levels [364].

In the case of nobiletin and luteolin, their administration to HFD-fed mice improved the fibrotic and inflammatory profile in adipose tissue and reduced the macrophage infiltration and polarization [344,346,365,366]; but in contrast with other flavones they increased the mRNA expression of FAO- (*Pparα*, *Cox8b*, and *Cpt1a*) and lipogenic (*Pparγ*, *Srebp1c, Fasn* and *Scd1)* -related genes simultaneously [342,344] as well as CPT1 and FASN activity [344] in WAT. The simultaneously activation of both metabolic pathways in adipose tissues has been demonstrated as a way to maintain thermogenesis in BAT [367,368] and as a marker of browning in WAT [82]. In the case of luteolin, its administration either in HFD-fed or low-fat-fed mice activated browning and thermogenesis in mice via the AMPK/PGC1α cascade. Under the AMPK/PGC1α signal, luteolin increased energy expenditure in HFD-fed mice and upregulated the mRNA levels of *Pgc1α, PPARα, Cidea* and *Sirt1* in BAT as well as *Ucp1 Pgc1α, Tmem26, Cidea, PPARα, Sirt1, Elovl3 and Cited1* in scWAT [369]. Moreover, the increased of PPAR*γ* protein levels in WAT has been linked to an alleviation of the hepatic lipotoxicity in HFD-fed mice [347]. Similar effects were observed with baicalein that administered to HFD-fed mice decreased pP38MAPK, pERK and PPARγ levels and increased pAKT, PGC1α and UCP1 as well as the presence of GLUT4 in cell membranes of the eWAT. Globally, baicalein reversed the glucose intolerance and insulin resistance produced by HFD [370].

Besides the effects of each compound by itself some flavones-rich extracts or foods or combinations of different bioactive compounds have been evaluated regarding their potential therapeutic role against obesity and its metabolic and inflammatory features [371,372].

### 8.3. Flavones and Obesity in the CNS: No Clear Evidences

There are few studies describing the potential role of flavones in obesity-related central alterations. Just luteolin has been demonstrated a protective effect against HFD-induced cognitive effects in obese mice. Luteolin administration alleviated neuroinflammation, oxidative stress and neuronal insulin resistance as well as improved the Morris water maze (MWM) and step-through task and increased the levels of BDNF [373]. Other effects of flavones described recently are anxiolytic-like activity [374], neuroprotection against gamma-radiation [375] treatment of glioblastoma [376], amelioration of the hypoxia-reoxygenation injury [377] or inhibition of the neuroinflammation caused by LPS [378].

## 9. Chalcones

Chalcones is a group of polyphenolic compounds with a broad structural diversity. Chalcones are precursors of other flavonoids and responsible for the golden yellow pigments found in flowers, fruits, vegetables, spices, teas and different plant tissues. Although their metabolism in the gastrointestinal tract and their rate of absorption are not still completely known, chalcones have shown a wide variety of biological activities. Several studies have demonstrated that, either from natural sources or synthetic, chalcones can impact on glucose and lipid metabolism and their health benefits have been studied in relation to type 2 diabetes [379]. Chalcones have shown hypoglycemic capacity, the ability to modulate food intake and activate AMPK, as well as antioxidant, anti-inflammatory, anticancer, anti-obesity, hepatoprotective and neuroprotective properties [380,381,382,383,384,385,386,387,388,389,390,391,392] Although there are no many studies in humans the effects of chalcones in the obese phenotype in animal models are similar to the ones described for other flavonoids, thus suggesting a potential therapeutic role of these group of bioactive compounds.

### 9.1. The Hepatoprotective Role of Chalcones

Chalcones have hepatoprotective properties in NAFLD, alcoholic fatty liver, drug- and toxicant-induced liver injury, and liver cancer [381]. It has been described that chalcones are able to inhibit the synthesis of triglycerides and the lipogenesis, to increase FAO, and to modulate adiponectin production and signaling.

Licochalcone F, a novel synthetic retrochalcone, has shown anti-inflammatory properties when administered to diet-induced obese mice. Licochalcone F inhibited TNFa-induced NF-kB activation and the mRNA expression of several pro-inflammatory markers. In the liver licochalcone F alleviated hepatic steatosis, by decreasing lipid droplets and glycogen deposition [380]. On its side, Licochalcone A, a chalcone isolated from *Glycyrrhiza uralensis*, administered to HFD-fed mice, reduced body weight, decreased serum triglycerides, LDL free fatty acids and fasting blood glucose, ameliorated hepatic steatosis, reduced lipid droplet accumulation [393]. In the liver, licochalcone A downregulated the protein levels of SREBP1c, PPARγ, and FASN as well as increased the phosphorylation of HSL, ATGL and ACC enzymes [393]. Moreover, licochalcone A increase the protein levels of CPT1A and stimulated SIRT1 and AMPK activity [393]. Taken together, licochalcone A ameliorated obesity and NAFLD in mice at least in part by reducing the fatty acid synthesis and increasing lipolysis and FAO via the activation of the SIRT1/AMPK pathway.

In a mouse model of HFD-induced obesity, *trans*-chalcone reduced the ALT levels and increased the HDL [394]. Similarly, in a mouse model of non-alcoholic steatohepatitis KK-Ay mice, xanthohumol, the chalcone from beer hops (*Humulus lupulus* L.), diminished hepatic inflammation and prevented from the expression of profibrogenic genes in the liver [395] as well as lowered hepatic fatty acid synthesis through the downregulation of *Srebp1c* expression and promoted FAO by upregulating the mRNA expression of *Pparα* in KK-Ay mice [396]. Moreover, in HFD-fed mice, xanthohumol prevented body weight gain; decreased glycemia, triglyceride and cholesterol, and improved insulin sensitivity. Xanthohumol activated the hepatic and skeletal muscle AMPK, downregulated the expression of *Srebp1c* and *Fasn* and inhibited the activity of ACC, thus reducing the lipogenic pathway [386,397].

According to these data, aspalathin a C-glucosyl dihydrochalcone present in rooibos tea from *Aspalathus linearis,* also activated AMPK and reduced the expression of hepatic enzymes and transcriptional regulators that are associated with either gluconeogenesis and/or lipogenesis (*Acc, Fasn, Scd)* in diabetic *ob/ob* mice [388,398]. Furthermore, Aspalathin-enriched green rooibos extract (GRE) improved hepatic insulin resistance via the regulation of the PI3K/AKT and AMPK Pathways [399]. In obese insulin resistant rats GRE upregulated the expression of *Glut2*, *insulin receptor (Insr), Irs1* and *Irs2*, as well as *Cpt1a* [399]. Finally, Isoliquiritigenin at a low dose ameliorated insulin resistance and NAFLD in diet-induced obese mice. Isoliquiritigenin administration to HFD-fed mice decreased body fat mass and plasma cholesterol as well as alleviated hepatic steatosis (smaller lipid droplets) with no changes in TG and FFA serum levels [400]. It has been described that isoliquiritigenin suppressed the expression of lipogenic genes (*Fasn* and *Scd1*) and increased FAO activity. Moreover, isoliquiritigenin improved the insulin signaling in the liver and muscle [400].

Besides chalcones, chalcones-enriched products like Safflower yellow or ashitaba have demonstrated hepatoprotective properties. In mice fed with HFD, Safflower yellow improved lipid profile and alleviated fatty liver in a mechanism that has been associated to a reduction of the biosynthesis of intracellular cholesterol. Safflower yellow significantly reduced the levels of total cholesterol, triglycerides, LDL-cholesterol and the LDL/HDL ratio [401]. On its side, ashitaba (*Angelica keiskei)* extract showed hepatoprotective activity in fructose-induced dyslipidemia due to increased expression of FAO genes in the liver. Treatment with this extract upregulated the expression of the *Acox1, Mcad, ATP-binding membrane cassette transporter A1 (ABCA1)* and *apolipoprotein A1 (Apo-A1)* [402]. In a similar way, this extract exerted hepatoprotective effects in HFD-fed mice. Ashitaba extract reduced plasma levels of cholesterol, glucose, and insulin, lowered triglyceride and cholesterol content in the liver, inhibited hepatic lipogenesis by downregulating *Srebp1* and *Fasn* and activated FAO by upregulating the expression of *Cpt1A* and *Pparα* [403]. The proposed mechanism underlying this hepatic metabolic effects is an activation of the AMPK enzyme in the liver [403].

In some of the studies the hepatoprotective role of chalcones has been linked to the adiponectin production. Concretely, trans-chalcone administration to high cholesterol diet-induced liver fibrosis increased the serum levels of adiponectin and the hepatic antioxidant enzymes, thus alleviating liver damage [404]. Similarly, xanthohumol and ashitaba extract or licochalcone A also increased the adiponectin expression and secretion [393,403,405].

### 9.2. Chalcones in the Adipose Tissue, Upregulation of Adiponectin, Induction of Browning and Enhancement of Energy Expenditure

As has been mentioned above, chalcones induce adiponectin expression and secretion but also improve adipocytes function and reduce fat depots. Different molecular mechanisms underlying these effects has been described.

The treatment of obese mice with licochalcone F to reduced adipocyte size and ameliorated macrophage infiltration in WAT depots as well as enhanced Akt signaling and reduced p38 MAPK pathway [380]. On its side, the administration of Licochalcone A, isoliquiritigenin or a *Glycyrrhiza uralensis* extract containing licochalcone A, isoliquiritigenin, and liquiritigenin to diet-induced obese mice reduced body weight gain and adipose tissues depots [393,400,406]. In this case, Licochalcone A and *Glycyrrhiza uralensis* extract induced the browning phenotype in the iWAT this fat depot [393,406] as it is demonstrated by the enhanced expression of brown fat markers such as *Ucp1, Prdm16* and *Pgc1α* [406]. By contrast, isoliquiritigenin elevated energy expenditure by increasing the expression of thermogenic genes (*Ucp1* and *Prdm16*) as well as *Sirt1* that is linked to mitochondrial biogenesis [407] in interscapular BAT [400].

Finally, butein, besides its anti-inflammatory activity via the p38 MAPK/Nrf2/HO-1 pathway that leads to a reduction of the adipocyte hypertrophy [408] is also capable to enhance energy expenditure and increase thermogenesis. Butein induced the browning phenotype in the iWAT (upregulation of *Ucp1, Prdm16, cytochrome C oxidase 8b,* and *Cidea)* and increased the UCP1 protein levels in BAT in HFD-fed mice as well as in lean mice. The proposed molecular mechanism underlying these effects is the induction of the PR domain containing 4 (Prdm4) and the activation of the PI3Kα/Akt1/PR domain containing 4 (Prdm4) axis [409,410]. The browning effect of butein was not observed in other mice models such as ThermoMouse strain nor in methionine- and choline-deficient diet-fed mice [411]. Butein actions have also been linked to its capacity to downregulate PPARγ expression [387,410].

Finally, chalcone-rich extracts such as Safflower yellow or Ashitaba extract have also demonstrated effects in adipose tissues. Concretely, in mice fed with HFD, Safflower yellow administration exerts anti-obesity and insulin-sensitizing effects by upregulating the expression of *Pgc1α* that may indicate a browning phenotype of the scWAT as well as activating the protein levels of AKT and GSK3β in visceral WAT [412]. On its side, Ashitaba extract suppressed the HF diet-induced body weight gain and fat deposition in WAT, increased the adiponectin level and the phosphorylation AMPK, inhibited lipogenesis by downregulating *Pparγ, CCAAT/enhancer-binding protein α (C/ebpα)* and *Srebp1* [403].

### 9.3. Chalcones in CNS: A Potential Neuroprotective Role

The antioxidant and anti-inflammatory properties of chalcones has been linked to some of their neuroprotective effects [382,383,389] but no studies with obesity-related neuronal damage has been found. Further studies are needed to identify the potential therapeutic role of chalcones on this obesity side effect.

## 10. Concluding Remarks

**Figure 1:**
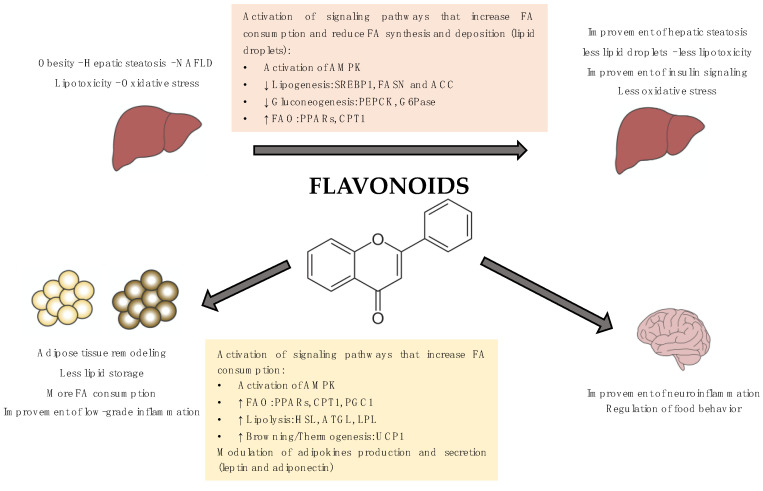
Summary of the metabolic and signaling pathways underlying the anti-obesity effects of flavonoids. Molecular mechanisms underlying the beneficial effects of flavonoids have been widely studied and, in many cases, involved the activation of the AMP-activated protein kinase (AMPK). AMPK is a key enzyme for the control of lipid metabolism and adipogenesis. AMPK phosphorylation and activation promote catabolic processes such as FAO, glucose uptake, or glycolysis as well as inhibits anabolic pathways such as fatty acid synthesis or gluconeogenesis.

Undoubtedly flavonoids are potential therapeutic agents against metabolic disorders such as obesity, type 2 diabetes or NAFLD. Their impact in CNS, liver, and adipose tissue has been extensively studied and the results let us to be optimistic. Several metabolic effects and signaling pathways have been described underlying the anti-obesity effects of flavonoids specially in liver, EAT and BAT but also in CNS. Globally theses effects go to control body weight, improve insulin sensitivity, reduce fat accumulation in adipose tissues as well in ectopic depots and to increase energy expenditure (Figure 1). Furthermore, the data presented in this review highlight that:

- Flavonoids are effective over a high variety of obesity and obesity-related diseases models.
- The anti-obesity effects of flavonoids are robust and consistent as they can be achieved using different sources, ways of administration and doses.
- Most of the molecular mechanisms underlying the anti-obesity effects of flavonoids are shared for the different subclasses of flavonoids (Table 2 and Table 3).

Even so more research is needed to confirm their therapeutically functionality in humans, the doses and times needed for their effectiveness or the better combination of bioactive compounds. Nowadays is still difficult to answer some crucial questions such as what is the effective dose of polyphenols; and for how long do we need to intake them to get positive effects? It is obvious that differences among experimental diets to induce fatty liver, dosages of bioactive compounds as well as the presence of other food compounds or the use of isolated or extracted polyphenols could influence the outcomes obtained. Furthermore, the use of flavonoids as a preventive or for treatment also show different results. Usually, the doses used in published papers are much higher than the ones reached from fruits and vegetables consumed as a whole.

The Predimed study determined that Spanish adults should intake around 820 ± 323 mg of polyphones/day in a 2000 Kcal diet to get their beneficial effects [25,27] but probably these effects at this dose are closely related to the MedDiet lifestyle. It is evident that, as MedDiet, some other dietary patterns include high amounts of fruits, vegetables or polyphenols-rich beverages that make possible to reach the optimal doses of polyphenols and by extension of flavonoids. Then, the question is: Are the effects of polyphenols linked to the dietary pattern where they are included? Two recent systematic reviews analyzed if there are enough evidence to define a health promoting polyphenol-rich dietary pattern and concluded that the high variability in the experimental approaches and methods used to evaluate polyphenols intake and health outcomes make difficult to stablish specific polyphenol intake recommendations and to clarify whether total flavonoids or rather individual subclasses may exert beneficial effects [30,36].

Moreover, low is known about the effects of combining different bioactive compounds from different families. Are they going to have synergic, additive or antagonic effects? And not less important is the need to identify the role of the food matrix on polyphenols and flavonoid effects.

The bioavailability of polyphenols is low and not just their basic chemical structures (aglycons) are key but also the attachment of additional groups. There are described around 8000 structures of polyphenols with different physiological impact and several chemical structures, but all of them with at least one a phenolic ring with one or more hydroxyl groups attached [38,413,414]. The polyphenols absorption in human body is dose- and type-dependent and their effects are related to their bioavailability and pharmacokinetics. They show a low absorption rate and limited stability during pass through the intestinal tract where microbiome may contribute to their absorption. Once absorbed, polyphenols enter portal circulation and are metabolized in the liver. This first pass metabolism modifies the polyphenol structure and in consequence its bioavailability and bioactivity [415,416]. Finally, the conjugate metabolites reach the bloodstream and the target tissues [415,416,417,418].

Several studies have demonstrated that the bioavailability and safety of polyphenols changed when they are included in a food matrix [419,420,421]. Although most of the assays has been done with in vitro models of digestion [422] it seems that the food matrices protect bioactive compounds from intestinal degradation [420,423]. Finally, also cooking processes would have an impact in the polyphenols content and bioavailability of some preparations [424,425,426]. On the other side, it has been described that bioactive compounds with antioxidant properties are safe and beneficial but that exogenous supplementation with isolated compounds can be toxic [427].

The role of intestinal digestion and microbiota impact on polyphenols’ effects must be also considered. Besides their direct action in the liver, some flavonoids may exert their metabolic effects through the gut microbiota modulation. An experimental approach with rabbits described that procyanidin b2 may downregulated fatty acid synthesis genes and protected against obesity and NAFLD by increasing the ratio of *Bacteroidetes* and *Akkermansia* [159]. Similar results were obtained with green tea oolong tea and black tea water extracts that administered to HFD-fed mice improved the glucose tolerance and reduced the weight gained caused by the HFD. Moreover, these animals showed a better hepatic lipid profile and a reduced mass of the WAT. These effects were accompanied by a reduction in plasma LPS, thus indicating less production and a significant increase in the production of short-chain fatty acids (SCFAs). A metagenomic analysis indicated that the tea extracts changed the gut microbiota’s composition [428]. In the same way also flavones ‘effects on obesity has been linked to gut microbiota modifications [338]. Oral hydroxysafflor yellow A (HSYA) reversed the HFD-induced gut microbiota dysbiosis and reduced the obese phenotype [429].

## Figures and Tables

**Table 1:** Flavonoids subclasses: compounds, representative food sources and chemical structures.

Compounds	Representative Food Source	Subclass	Chemical Structure
Cyanidin Delphinidin Malvidin Peonidin	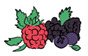	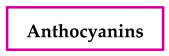	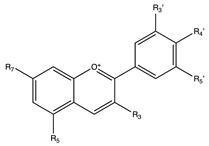
(+)-Catechin (−)-Epicatechin (−)-Epigallocatechin (−)-Epigallocatechin gallate Procyanidin dimer B2	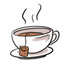	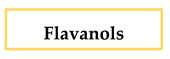	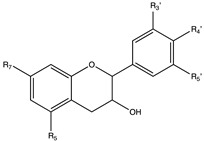
Hesperetin Hesperidin Naringenin Naringin Eriodyctiol	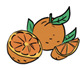	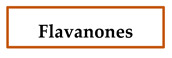	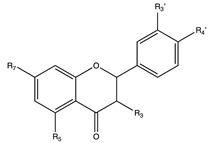
Kaempferol Myricetin Quercetin Isoquercetin	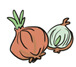	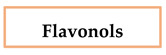	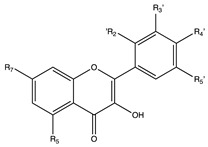
Daidzein Genistein	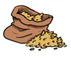	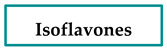	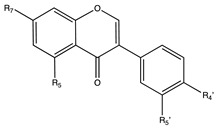
pigenin Chrysin Luteolin Baicalin Vitexin Nobiletin	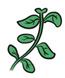	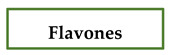	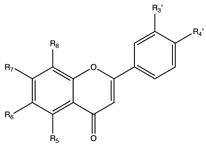
Butein Licochalcone Isoliquiritigenin Xanthohumol	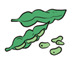	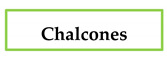	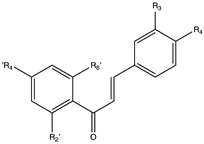

**Table 2:** Metabolic effects and signaling pathways underlying the anti-obesity effects of flavonoids in the liver.

Liver	Anthocyanins	Flavanols		Flavanones		Flavonols
**Signaling pathways**	Activation of the AMPK Inactivation of mTOR pathway	Activation of the AMPK. Activation of SIRT and SIRT/FoxO1 pathway. Activation of the PKB/AKT—p-GSK3a and p-PTEN Activation of PKA Inactivation of mTOR pathway Activation of the PXR/CAR-mediated phase II metabolism		Activation of the AMPK Activation of AMPK/SIRT1/PGC1α axis Activation of FGF21 signaling		Activation of the AMPK Activation of AMPK/PG1α/ERRα axis Inactivation of LXR/SREBP1c axis Inactivation of mTOR pathway Inhibition of the PKB/AKT—downregulation of SREBP1 ↑ Adiponectin signaling
**Lipid metabolism**	↓ Lipogenesis and TG synthesis ↑ FA consumption (FAO) ↓ Lipid droplets	↓ Lipogenesis and TG synthesis ↑ FA consumption (FAO) ↓ Lipid droplets ↓ Cholesterol synthesis and bile acids reabsorption		↓ Lipogenesis and TG synthesis ↑ FA consumption (FAO) ↓ Lipid droplets		↓ Lipogenesis and TG synthesis ↑ FA consumption (FAO) ↓ Lipid droplets ↓ Cholesterol synthesis
**Glucose metabolism**	↓ Gluconeogenesis ↑ Glucose transport ↑ Glycolysis ↑ Insulin signaling	↓ Gluconeogenesis		↓ Gluconeogenesis ↓ Glucose transport ↑ Glycolysis		↑ Glucose transport ↑ Insulin signaling
**LIVER**	**Isoflavones**		**Flavones**		**Chalcones**	
**Signaling pathways**	Activation of the AMPK Blockade of aldose reductase (AR)/polyol pathway Activation of the PKB/AKT		Activation of the AMPK. Inactivation of LXR/SREBP1c axis. Nuclear erythroid 2-related factor 2 (Nrf2) and PPARγ activity ↑ Adiponectin signaling		Activation of the AMPK Activation of AMPK/SIRT pathway Activation PI3K/AKT/PRDM4 signaling	
**Lipid metabolism**	↓ Lipogenesis and TG synthesis ↑ FA consumption (FAO) ↓ Cholesterol synthesis		↓ Lipogenesis and TG synthesis ↑↓ FA consumption (FAO)		↓ Lipogenesis and TG synthesis ↑ FA consumption (FAO) ↓ Lipid droplets ↓ Cholesterol synthesis	
**Glucose metabolism**	↓ Gluconeogenesis ↑ Glucose transport ↑ Insulin signaling		↓ Gluconeogenesis		↓ Gluconeogenesis ↑ Glucose transport ↑ Insulin signaling	

**Table 3:** Metabolic effects and signaling pathways underlying the anti-obesity effects of flavonoids in the adipose tissues.

Adipose Tissue	Anthocyanins	Flavanols		Flavanones		Flavonols
**Signaling pathways**	Activation of the AMPK Activation of SIRT and SIRT/FoxO1 pathway Activation of the FDNC5/Irisin pathway ↑ FGF21 signaling	Activation of b-adrenergic receptor—↑ cAMP/PKA Inhibition of the PDE ↑ Adiponectin signaling				Activation of b-adrenergic receptor—↑ cAMP/PKA Activation of the AMPK/SIRT1 pathway Activation of SIRT1/PGC1α axis
**Adipokines**	↓ Leptin ↑ Adiponectin			↓ Leptin ↑ Adiponectin		↑ Adiponectin
**Adipose tissue profile**	↑ Browning and Thermogenesis ↓ Adipogenesis	↑ Browning and Thermogenesis ↓ Adipogenesis		↑ Browning and Thermogenesis		↑ Browning and Thermogenesis ↓↑ Adipogenesis
**Lipid metabolism**	↑ FA consumption (lipolysis and FAO) ↓↑ Lipogenesis and TG synthesis ↓ Lipid droplets	↑ FA consumption (lipolysis and FAO) ↓ Lipogenesis and TG synthesis		↑ FA consumption (lipolysis and FAO) ↓ Lipogenesis and TG synthesis		↑ FA consumption (lipolysis and FAO) ↓ Lipogenesis and TG synthesis
**Glucose metabolism**	↑ Glucose transport			↑ Glucose transport		↑ Glucose transport
**ADIPOSE TISSUE**	**Isoflavones**		**Flavones**		**Chalcones**	
**Signaling pathways**	Activation of the AMPK Activation of the FDNC5/Irisin pathway		Activation of the AMPK Activation of the AMPK/PGC1α axis Activation of the STAT3/CD36 signaling pathway		Activation of the AMPK Activation PI3K/AKT signaling	
**Adipokines**	↓ Leptin ↑ Adiponectin				↑ Adiponectin	
**Adipose tissue profile**	↑ Browning and Thermogenesis		↑ Browning and Thermogenesis ↓ Adipogenesis		↑ Browning and Thermogenesis	
**Lipid metabolism**	↑ FA consumption (lipolysis and FAO) ↓ Lipid droplets ↓ Lipogenesis and TG synthesis		↑ FA consumption (lipolysis and FAO) ↓ Lipid droplets ↓↑ Lipogenesis and TG synthesis		↑ FA consumption (lipolysis and FAO) ↓↑ Lipogenesis and TG synthesis	
**Glucose metabolism**			↑ Glucose transport			
